# A Highly Sensitive CRISPR‐Empowered Surface Plasmon Resonance Sensor for Diagnosis of Inherited Diseases with Femtomolar‐Level Real‐Time Quantification

**DOI:** 10.1002/advs.202105231

**Published:** 2022-03-27

**Authors:** Fei Zheng, Zhi Chen, Jingfeng Li, Rui Wu, Bin Zhang, Guohui Nie, Zhongjian Xie, Han Zhang

**Affiliations:** ^1^ Shenzhen Engineering Laboratory of phosphorene and Optoelectronics International Collaborative Laboratory of 2D Materials for Optoelectronics Science and Technology of Ministry of Education Shenzhen Institute of Translational Medicine Department of Otolaryngology Shenzhen Second People's Hospital the First Affiliated Hospital Institute of Microscale Optoelectronics Shenzhen University Shenzhen 518060 P.R. China; ^2^ Shenzhen International Institute for Biomedical Research Shenzhen 518110 China; ^3^ Laboratory of Robotics and System Harbin Institute of Technology Harbin 150001 P. R. China; ^4^ Institute of Pediatrics Shenzhen Children's Hospital Shenzhen Guangdong 518038 P. R. China; ^5^ Shenzhen International Institute for Biomedical Research Shenzhen Guangdong 518116 P. R. China

**Keywords:** biosensor, clustered regularly interspaced short palindromic repeats (CRISPR), nucleic acid detection, surface plasmon resonance, two‐dimensional materials

## Abstract

The clustered regularly interspaced short palindromic repeats (CRISPR) molecular system has emerged as a promising technology for the detection of nucleic acids. Herein, the development of a surface plasmon resonance (SPR) sensor that is functionalized with a layer of locally grown graphdiyne film, achieving excellent sensing performance when coupled with catalytically deactivated CRISPR‐associated protein 9 (dCas9), is reported. dCas9 protein is immobilized on the sensor surface and complexed with a specific single‐guide RNA, enabling the amplification‐free detection of target sequences within genomic DNA. The sensor, termed CRISPR‐SPR‐Chip, is used to successfully analyze recombinant plasmids with only three‐base mutations with a limit of detection as low as 1.3 fM. Real‐time monitoring CRISPR‐SPR‐Chip is used to analyze clinical samples of patients with Duchenne muscular dystrophy with two exon deletions, which are detected without any pre‐amplification step, yielding significantly positive results within 5 min. The ability of this novel CRISPR‐empowered SPR (CRISPR‐eSPR) sensing platform to rapidly, precisely, sensitively, and specifically detect a target gene sequence provides a new on‐chip optic approach for clinical gene analysis.

## Graphical abstract

This paper presents a surface plasmon resonance (SPR) based, femtomolar‐level, pre‐amplification‐free, real‐time genomic DNAs quantification platform "CRISPR‐eSPR". This unprecedented specificity and efficiency are achieved by the innovative marriage of the unique sequence‐specific recognizing ability of the clustered regularly interspaced short palindromic repeats (CRISPR)/Cas system and the superior sensitivity of the 2D‐material enforced Surface Plasmon Resonance sensor. The feasibility of clinical diagnosis has been strongly proved.

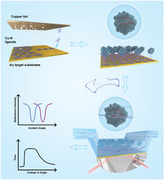

## 1 Introduction

The rapid development of whole‐genome sequencing has revolutionized human genetic studies.^[^ ^1^, ^2^ ^]^ In particular, targeted sequencing including all protein‐coding genes provides an opportunity for precise medical treatment.^[^ ^3^ ^]^ Polymerase chain reaction (PCR) and its derivatives are well‐established methods for the molecular evaluation and quantification of nucleic acids.^[^ ^4^, ^5^, ^6^, ^7^, ^8^, ^9^, ^10^ ^]^ However, these amplification‐dependent techniques are laborious, costly, and time‐consuming, requiring complex reactions using multiple reagents, skillful operators, and expensive equipment. Therefore, new techniques are urgently needed to circumvent the limitations of conventional nucleic acid detection and promote the development of an inexpensive and systematic gene targeting platform for amplification in clinical settings.

The clustered regularly interspaced short palindromic repeat (CRISPR) system, a natural adaptive immune system that evolved in bacteria and archaea, has been developed as a revolutionary genomic editing tool.^[^ ^11^ ^]^ Typically, the CRISPR‐associated 9 (Cas9) nuclease can be navigated by a chimeric single‐guide RNA (sgRNA) to any genomic locus using a 5′‐NGG protospacer‐adjacent motif to recognize a site‐specific double‐strand sequence.^[^ ^12^ ^]^ This discovery led to numerous research efforts associated with gene therapies in various fields.^[^ ^13^, ^14^ ^]^ Some gene detection methodologies use different Cas nucleases targeting various types of nucleic acid analytes. For example, Kellner et al. reported the SHERLOCK methodology employing Cas13, an RNA‐guided RNase, to detect amplified RNA sequences by recombinase polymerase amplification (RPA) with the introduction of the T7 RNA polymerase promoter.^[^ ^15^ ^]^ The HOLMES methodology reported by Li et al. integrates Cas12a (also known as Cpf1)^[^ ^16^ ^]^ and isothermal amplification to detect single‐stranded DNA with attomolar sensitivity.^[^ ^17^ ^]^ Further, HOLMESv2 introduces a combination of Cas12b (also known as C2c1) and loop‐mediated isothermal amplification to quantify RNA and identify single nucleotide polymorphisms.^[^ ^18^ ^]^ With the advantages of high specificity, sensitivity, and flexibility, these CRISPR‐based gene detection techniques have progressively been applied in various clinical scenarios, including the detection of bacteria,^[^ ^19^ ^]^ diagnosis of hereditary disease,^[^ ^20^ ^]^ and screening of viruses.^[^ ^21^ ^]^ However, these CRISPR‐based detection methodologies rely on the amplification of nucleic acids via PCR or RPA, which are time‐consuming processes,^[^ ^22^ ^]^ or require complicated processes for fabrication of the device, resulting in a high usage cost. Recently, researchers have avoided the use of polymerase‐mediated amplification in an attempt to improve sensitivity. For example, Tian et al. developed an amplification‐free CRISPR‐Dx system based on droplet microfluidics, achieving a confined RNA‐triggered Cas13a catalysis system in cell‐like‐sized reactors to enhance local concentrations of target and reporter genes.^[^ ^23^ ^]^ Furthermore, modular catalytic hairpin assembly circuits had been designed to enhance the sensitivity of CRISPR‐Dx reactions to detect unamplified miRNA and genomic DNA samples.^[^ ^24^, ^25^ ^]^

The delicate design of CRISPR‐Dx reactions described above combining different platforms for enhanced signals is also a great path to further improve the sensitivity of CRISPR‐Dx. Kim et al. developed a CRISPR‐mediated surface‐enhanced Raman scattering (SERS) assay to detect magnetic bead‐enriched genomic DNA of multidrug‐resistant bacteria, reaching a high sensitivity (≈8–14 fM).^[^ ^26^ ^]^ Hajian et al. reported a CRISPR‐coupled graphene field‐effect transistor, in which a catalytically deactivated Cas9 (dCas9) was anchored to a liquid‐gate electrode, serving as a specific capturer for the detection of unamplified clinical genomic DNA with femtomolar resolution.^[^ ^20^ ^]^

Surface plasmon resonance (SPR)‐sensing technology has been proven to be powerful and versatile in investigations of molecular interactions such as drug‐target,^[^ ^27^ ^]^ protein‐protein,^[^ ^28^ ^]^ protein‐nucleic acid,^[^ ^29^ ^]^ protein‐lipid,^[^ ^30^ ^]^ and antigen‐antibody,^[^ ^31^ ^]^ by monitoring the refractive index change on the sensor surface caused by the changed weight. Various two‐dimensional (2D) materials have been used to functionalize SPR sensors to improve the sensing performance, including graphene,^[^ ^32^ ^]^ antimonene,^[^ ^33^ ^]^ transition‐metal dichalcogenides,^[^ ^34^ ^]^ black phosphorus,^[^ ^35^ ^]^ and MXenes.^[^ ^36^ ^]^ However, most of these 2D materials are subject to limitations, including poor stability in physiological environments and challenging to form a uniform coating film. As a novel 2D carbon allotrope with a unique planar structure, graphdiyne has been successfully applied in many areas such as catalysis,^[^ ^37^ ^]^ energy,^[^ ^38^ ^]^ environment,^[^ ^39^ ^]^ and biomedicine,^[^ ^40^ ^]^ owing to its excellent properties in mechanics, electronics, and optics. Therefore, we were inspired to develop a CRISPR‐empowered SPR biosensor, taking advantage of its potentially super high sensitivity to detect the clinical genomic DNA with disease‐related deletions. We also aimed to obtain real‐time signal monitoring and reach high sensitivity without pre‐amplification steps.

Genomic DNA detecting technology is valuable for early screening of inherited diseases. Although disease‐associated genetic variants may have deleterious impacts on various protein functions and lead to severe health disorders, inherited diseases often remain undiagnosed until symptoms manifest at an older age, thereby missing the opportunity for timely interventions and management.^[^ ^41^ ^]^ Duchenne muscular dystrophy (DMD) is an X‐linked recessive neuromuscular disease caused by mutations in the dystrophin gene (DMD),^[^ ^42^ ^]^ resulting in insufficient levels of the cytoskeletal protein, and thus negatively affecting the strength and functionality of myofibers.^[^ ^43^ ^]^ DMD mutations, especially deletions, are frequently detected between exons 45–55 and exons 2–10,^[^ ^44^ ^]^ providing promising genetic detection sites for an early diagnosis. Therefore, genomic DNA from patients with a deletion in exon 47 or exon 7 was used as the template for the testing of clinical samples with our CRISPR‐empowered SPR (CRISPR‐eSPR) sensor.

Herein, we first attempted to exploit the synergistic merit of the sequence‐specific recognizing ability of the CRISPR/Cas system and the high sensitivity of the SPR sensor (termed CRISPR‐SPR‐Chip), demonstrating a biosensor with femtomolar‐level real‐time quantification of genomic DNAs related to an inherited disease in clinical samples. Advantages of the CRISPR‐SPR‐Chip include the 1) graphdiyne film that affords a better protein‐loading capacity, 2) enhancement of the SPR signal response was also enhanced via the electromagnetic field coupling effect of graphdiyne with the Au metal layer, 3) ability of the dCas9/sgRNA immobilized on the graphdiyne film on the CRISPR‐SPR‐Chip to distinguish and capture specific DNA sequences at low concentrations without pre‐amplification (less than 5 fM). 4) demonstrated clinical application of the CRISPR‐SPR‐Chip as shown by the sensitive and accurate detection of genomic DNA in patients with Duchenne muscular dystrophy. Above all, this novel CRISPR empowered SPR (CRISPR‐eSPR) sensor can facilitate precise, stable, sensitive, and reliable gene analysis of clinical samples.

## 2 Results and Discussion

### 2.1 Fabrication of the CRISPR‐SPR‐Chip

**Figure 1:**
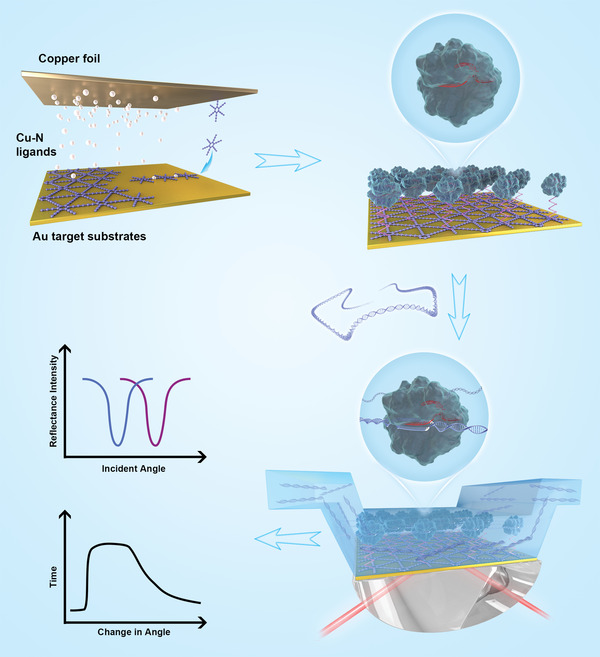
Schematic representation of the CRISPR‐SPR‐Chip sensing platform for the detection of target sequences within genomic DNA. A) Fabrication of an SPR sensor chip integrated with a graphdiyne film and the CRISPR/dCas9 system.

A schematic of the graphdiyne‐based construct of CRISPR‐SPR‐Chip is shown in **Figure** **1**A. A homogenous graphdiyne film was grown locally on the surface of the Au metal layer using the Glaser‐Hey coupling reaction of the hexaethynylbenzene monomer with the aid of copper catalysis.^[^ ^45^ ^]^ The as‐grown graphdiyne film was further functionalized with a molecular linker, PBASE, to attain the ‐NHS active group. The dCas9 nuclease was immobilized on the surface of the graphdiyne film via carbodiimide cross‐linking chemistry,^[^ ^46^ ^]^ followed by incubating with a specific sgRNA, forming the anchored dRNP complex.

The CRISPR‐SPR‐Chip utilizes the electromagnetic field coupling effect between the Au metal film and the 2D graphdiyne to enhance the SPR response signal. In addition, the high surface area feature of graphdiyne provides a high loading capacity for immobilization of the dCas9 nuclease. Therefore, the combination of graphdiyne and the CRISPR molecular system imparts a sensing performance to the novel CRISPR‐SPR‐Chip a sensing performance beyond that of the conventional bare Au‐based SPR chip.

### 2.2 Material Characterization

**Figure 2:**
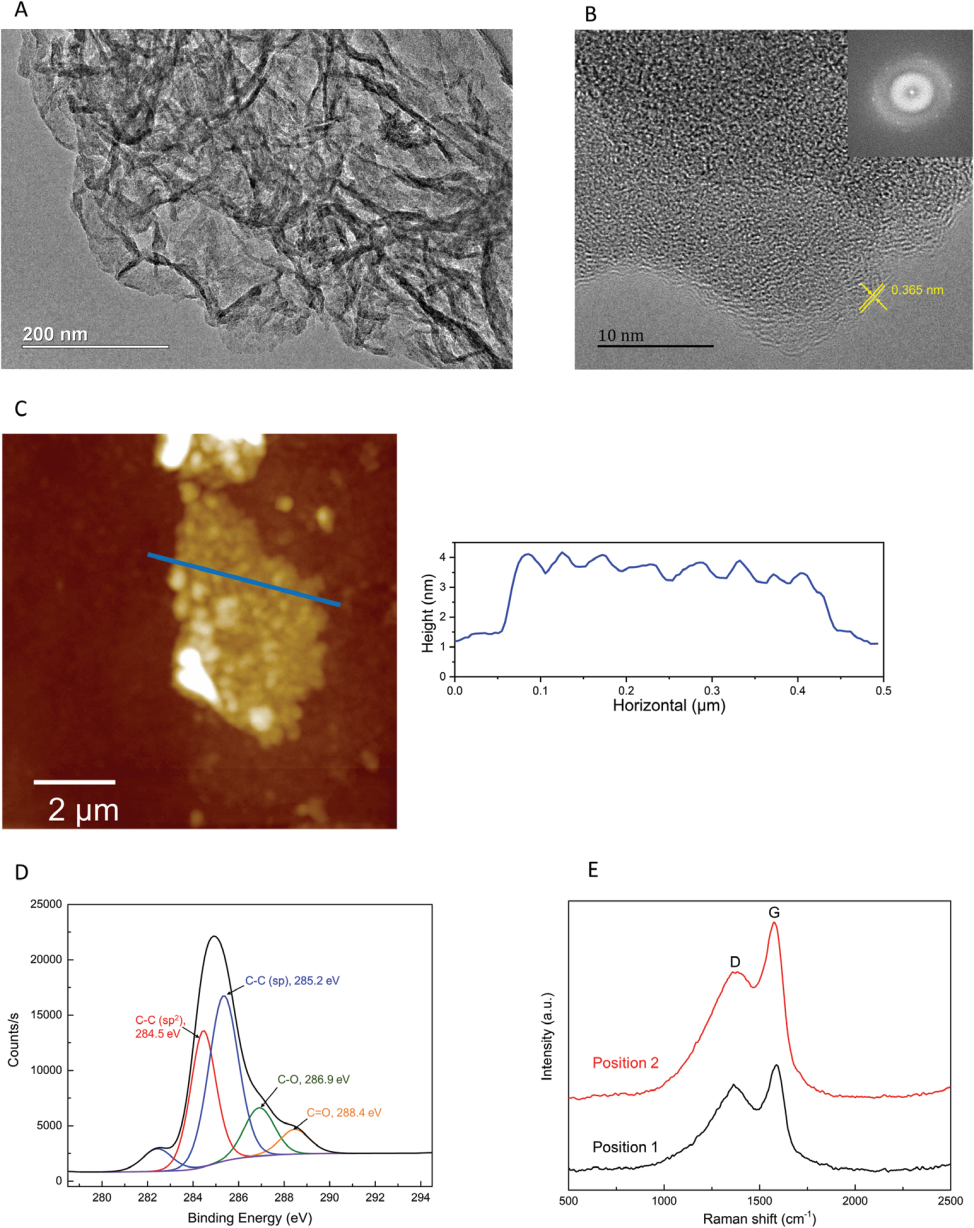
Characterization of the as‐prepared graphdiyne. A) TEM image of graphdiyne that was transferred onto a copper grid. B) High‐resolution TEM image of graphdiyne (inset is the fast Fourier transform image). C) AFM image (left) and the thickness histogram of the blue line (right). D) High‐resolution core‐level XPS spectra of C1s. E) Typical Raman spectra of graphdiyne.

The structure and elementary composition of the graphdiyne film grown on the SPR chips were characterized using transmission electron microscopy (TEM), atomic force microscopy (AFM), X‐ray photoelectron spectroscopy (XPS), and Raman spectroscopy, as described in the Supplementary Methods. The as‐prepared graphdiyne flakes showed unique 2D structural features by TEM (**Figure** **2**A). High‐resolution TEM (HRTEM) further revealed the 2D layered morphology of the graphdiyne film (Figure 2B). The interlayer gap spacing of graphdiyne was 0.365 nm, which corresponds to the calculated *π*‐stacking interlayer distance ranging from 0.34 to 0.37 nm with different numerical configurations.^[^ ^47^ ^]^ Fast Fourier transform of the HRTEM image in the inset of Figure 2B demonstrated the hexagonal carbon network of graphdiyne. The thickness of the graphdiyne film was 3–4 nm, according to the results of AFM (Figure 2C). The XPS spectrum of graphdiyne revealed a peak at 285.0 eV, which showed an essentially identical binding energy for the C1s orbital, that could be deconvoluted into four subpeaks at 284.5, 285.2, 286.9, and 288.4 eV, assigned to C―C (sp^2^), C―C (sp), C―O, and C=O, respectively (Figure 2D).^[^ ^48^ ^]^ The oxygen‐containing groups may have originated from oxidation of the terminal alkyne.

The Raman scattering spectrum contains fingerprint structural information, especially for Raman‐active diyne linkers arranged in the specific topology of graphdiyne. As shown in Figure 2E, two prominent peaks appeared at 1363.7 cm^−1^ and 1587.9 cm^−1^. The peak at 1363.7 cm^−1^ corresponds to the breathing vibration of sp^2^ carbon domains in aromatic rings, which is a hypsochromic shift compared to the *D* band of graphite.^[^ ^49^ ^]^ The peak at 1587.9 cm^−1^ corresponds to the first‐order scattering of *E*_2g_ mode observed for in‐phase stretching vibration sp^2^ carbon domains in aromatic rings, which is a redshift compared to the *G* band of graphite.^[^ ^50^ ^]^ The high intensity of the *D* band indicated the presence of structural defects and amorphous carbon in the graphdiyne sample.^[^ ^50^ ^]^

### 2.3 Target DNA and sgRNA for the CRISPR Reaction

Successful site‐directed mutagenesis was verified by Sanger sequencing ( Figure S5, Supporting Information). Next, we used the wild‐type pUC19 vector and the mutated plasmids as PCR templates to produce DNA fragments, referred to as pUC19‐Origin, pUC19‐Mut1, pUC19‐Mut2, and pUC19‐Mut3. Since the length of the target DNA sequence may also influence the binding efficiency of the RNPs and dRNPs,^[^ ^51^ ^]^ fragment amplicons of 320 bp and 853 bp were obtained via PCR and named with the suffixes “−0.3k” and “−0.8k”, respectively (e.g., pUC19‐Origin‐0.3k).

### 2.4 Specificity Assay

**Figure 3:**
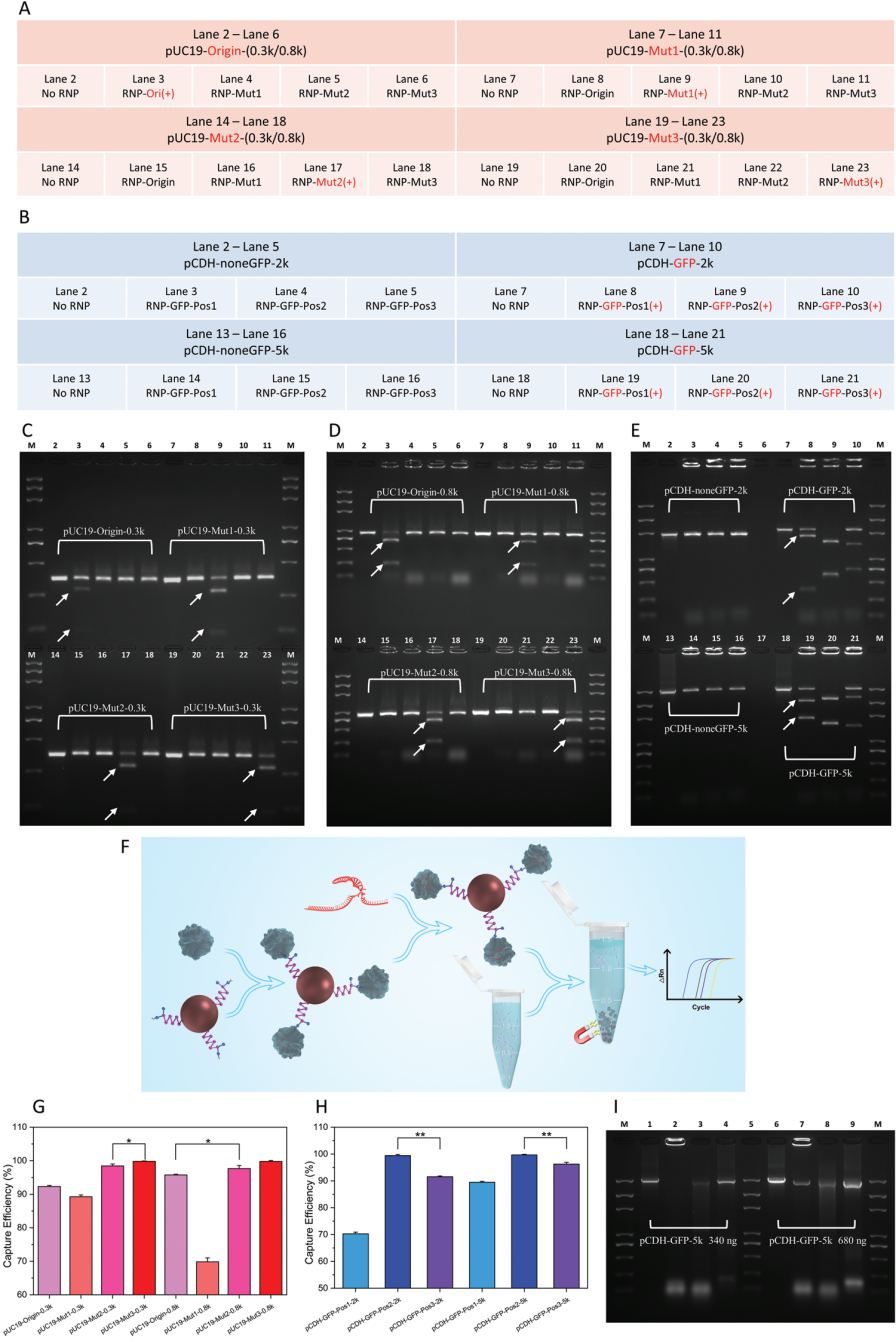
Specificity and efficiency evaluation of the CRISPR molecular system with pUC19 mutagens and the pCDH *GFP* gene. A) and B) Detailed information of all DNA‐RNP possible combinations for pUC19 mutated amplicons and pCDH amplicons analyzed by agarose gel electrophoresis. C) and D) TAE agarose gel electrophoresis analysis of CRISPR‐cleavage products for the pUC19‐0.3k and pUC19‐0.8k groups, related to Figure 3A. E) TAE agarose gel electrophoresis of CRISPR‐cleavage products for the pCDH‐2k and pCDH‐5k groups, related to Figure 3B. F) In vitro binding assays for evaluating the efficiency of CRISPR/dCas9 using CRISPR‐magnetic beads (MBs). G, H) Capture efficiency of different target sites calculated using the qPCR Ct results from Figure S6, Supporting Information, (*p < 0.05, **p < 0.01, two‐tailed *t*‐test, *n*  =  3; error bars represent standard deviations). I) TAE agarose gel electrophoresis analysis evaluate the capture efficiency of *GFP* DNA samples using dRNP‐functionalized beads and treated with different post‐release methods. A DNA molecular weight marker ranging from 100 bp to 5000 bp was applied in all agarose electrophoresis runs.

An in vitro cleavage assay was carried out to validate the specificity of the CRISPR molecular system. Four types of pUC19 PCR amplicons with nucleobase mutations and their corresponding RNPs were assayed using all possible combinations. Detailed information, including the correct DNA‐RNP combinations, are highlighted in red bold and labeled (+) in **Figure** **3**A. Electrophoresis of the CRISPR‐cleavage products revealed the fragment lengths of 320 bp and 853 bp (Figure 3C,D). In both the −0.3k and −0.8k groups, new bands were only observed in Lanes 3, 9, 17, and 23 (arrows), where the mutated pUC19 genes reacted with the corresponding RNPs. No cleaved DNA fragment was observed in any mismatch condition, indicating that the CRISPR molecular system can recognize the target sequences by specific RNPs without any detectable cross‐reactivity. Cleaved samples displayed shorter bands in accordance with the design of the target DNA and sgRNA. This indicated that the RNPs specifically bound to the target DNA sites during the CRISPR‐cleavage reaction. Because the DNA samples were loaded in identical amounts, a comparison of the band brightness of uncleaved and cleaved samples suggested that pUC19‐Mut3 exhibited the highest cleavage efficiency, which was confirmed by qPCR.

### 2.5 Efficiency Evaluation

To investigate the efficiency of different sgRNA sequences, three sgRNAs targeting different positions of the pCDH*‐GFP* gene were employed in another in vitro cleavage assay. As illustrated in Figure 3B, DNA amplicons with the accordant *GFP*‐specific RNPs are in red bold and labeled (+). As demonstrated in Figure 3E, new bands were observed in Lanes 8, 10, 19, and 21 (arrows), and no cleaved fragment was observed in the negative control (Lanes 2, 5, and 13–16). The length of each cleavage fragment precisely matched the pre‐designed target sites, indicating that both pCDH‐GFP‐2k and pCDH‐GFP‐5k were cleaved at the specified positions by their corresponding RNPs. Furthermore, the prominent cleavage efficiency of GFP‐Pos2 (Lane 9 and Lane 20) was demonstrated by the loss of the amplicon band and the brightest bands of the cleavage products. Based on the brightness of the bands, the binding efficiency among the three different sgRNAs was roughly estimated to be in the order GFP‐Pos2 > GFP‐Pos1 > GFP‐Pos3, which was confirmed by qPCR analysis.

### 2.6 In Vitro Binding Assays

Although the cleavage assays confirmed the specificity and efficiency of the CRISPR/Cas 9 system, the ultimate goal in using the CRISPR‐SPR‐Chip is to detect its target dsDNA by capturing specific sequences. Therefore, the capability of immobilized dRNPs to bind and maintain their affinity to the target DNA sequences was further evaluated with in vitro binding assays using MBs.

In Figure S6A,B, Supporting Information, the amplification curve closest to the left side (with the lowest Ct value) indicates the highest DNA concentration. The reference curve is reasonably on the left because the empty dCas9‐functionalized MBs adsorb very few amplicons (with negligible affinity), leading to the highest supernatant DNA concentration. In the case of pUC19, the amplification curves from left to the right are pUC19‐Mut1, pUC19‐Origin, pUC19‐Mut2, and pUC19‐Mut3, revealing that pUC19‐Mut3 dRNP had the highest affinity to its corresponding DNA fragment. The amplification curves of pCDH‐GFP‐2k (Figure S6C) and pCDH‐GFP‐5k (Figure S6D, Supporting Information) showed affinity in the order GFP‐Pos2 > GFP‐Pos1 > GFP‐Pos3, consistent with the in vitro cleavage assays (Figure 3C–E).

The workflow of capture efficiency measurement of pUC19 dRNPs is described in Figure 3F and the “method” section, and the results are summarized in Figure 3G based on the results from Figure S6A,B, Supporting Information. The highest capture efficiency of pUC19 was found with ‐Mut3 groups, which was 99.80% for pUC19‐Mut3‐0.3k and 99.79% for pUC19‐Mut3‐0.8k. The ‐Mut1 groups exhibited the lowest capture efficiencies (89.25% for pUC19‐Mut1‐0.3k and 69.83% for pUC19‐Mut1‐0.8k). Similarly, the capture efficiencies of pCDH dRNPs (Figure 3H) were calculated from the results in Figure S6C,D, Supporting Information. GFP‐Pos2 dRNPs exhibited the highest capture efficiencies (99.43% for ‐2k and 99.68% for ‐5k), which were significantly higher than those of GFP‐Pos3 and GFP‐Pos1, respectively (both *p* < 0.01). In the optimized condition, the dRNPs of ‐Mut3 for pUC19 and ‐Pos2 for pCDH bound to almost the entire corresponding amplicon molecules (nearly 100% capture efficiency). Therefore, these two dRNPs were subsequently used for further evaluation of the limit of detection (LOD) with our CRISPR‐SPR sensing platform.

### 2.7 In Vitro Capture/Release Assay of the Target DNA Fragments

To further understand the capture/release feature of dCas9 nuclease, we used the ‐Pos2 dRNP to capture the pCDH‐GFP‐5k amplicon and tested various post‐release treatments, including heat denaturation and enzymatic hydrolysis. As shown in Figure 3I, negative controls (Lanes 1 and 6) with 340  and 680 ng of pre‐incubation amplicons, respectively, displayed single bands at ≈5 kb. For control experiments without subsequent release treatment, the dRNP‐DNA complex was confined in the slot of the gel when the DNA fragment was 340 ng (Lane 2), indicating that a tight and steady combination of the dRNP and DNA was formed. Notably, when the amount of DNA was doubled (680 ng), a new band was observed in Lane 7 (≈5 kb), reflecting the excessive amount of DNA over the maximum loading capacity of the dRNP. With heat treatment (85 °C for 10 min) of the post‐incubation products of the dRNP‐DNA complex to denature the dCas9 nuclease, vague DNA bands at ≈5 kb were observed (Lanes 3 and 8), implying that heat denaturation partially released the captured DNA fragments. Finally, application of proteinase K to digest the dCas9 protein resulted in bands at ≈5 kb (Lanes 4 and 9) with almost the same brightness as that of the negative control, demonstrating full release of the DNA after enzymatic hydrolysis. These results further proved that the dCas9 protein can capture the target dsDNA and form a stable complex in the presence of specific sgRNA. Furthermore, heat denaturation and enzymatic hydrolysis of dCas9 protein led to the release of the captured dsDNA, demonstrating that this capture behavior is reversible.

### 2.8 SPR Measurement

A higher loading amount of dCas9 protein and the sgRNA was observed on the graphdiyne‐modified SPR chip than on the bare chip without functionalization (Figure S8A,B, Supporting Information). At a concentration of 250 ng µL^−1^ dCas9 protein, the graphdiyne‐modified chips were saturated, whereas the bare chips were saturated at a concentration as low as ≈150 ng µL^−1^ (Figure S8A, Supporting Information). Also, the saturated loading amount of sgRNA on the graphdiyne‐modified SPR chip was higher than that of the bare chip (Figure S8B, Supporting Information, 1.70 µg vs. 1.21 µg), benefiting from the higher dCas9 protein loading. In the following sensing investigation, the loading concentration of dCas9 and sgRNA were fixed at their saturation point (250 ng µL^−1^). Meanwhile, the immobilization of dCas9 and sgRNA on the chips are verified using immunological methods, and agarose gel electrophoresis (Verification of the immobilization of dCas9 and sgRNA, and Figure S9, Supporting Information).

**Figure 4:**
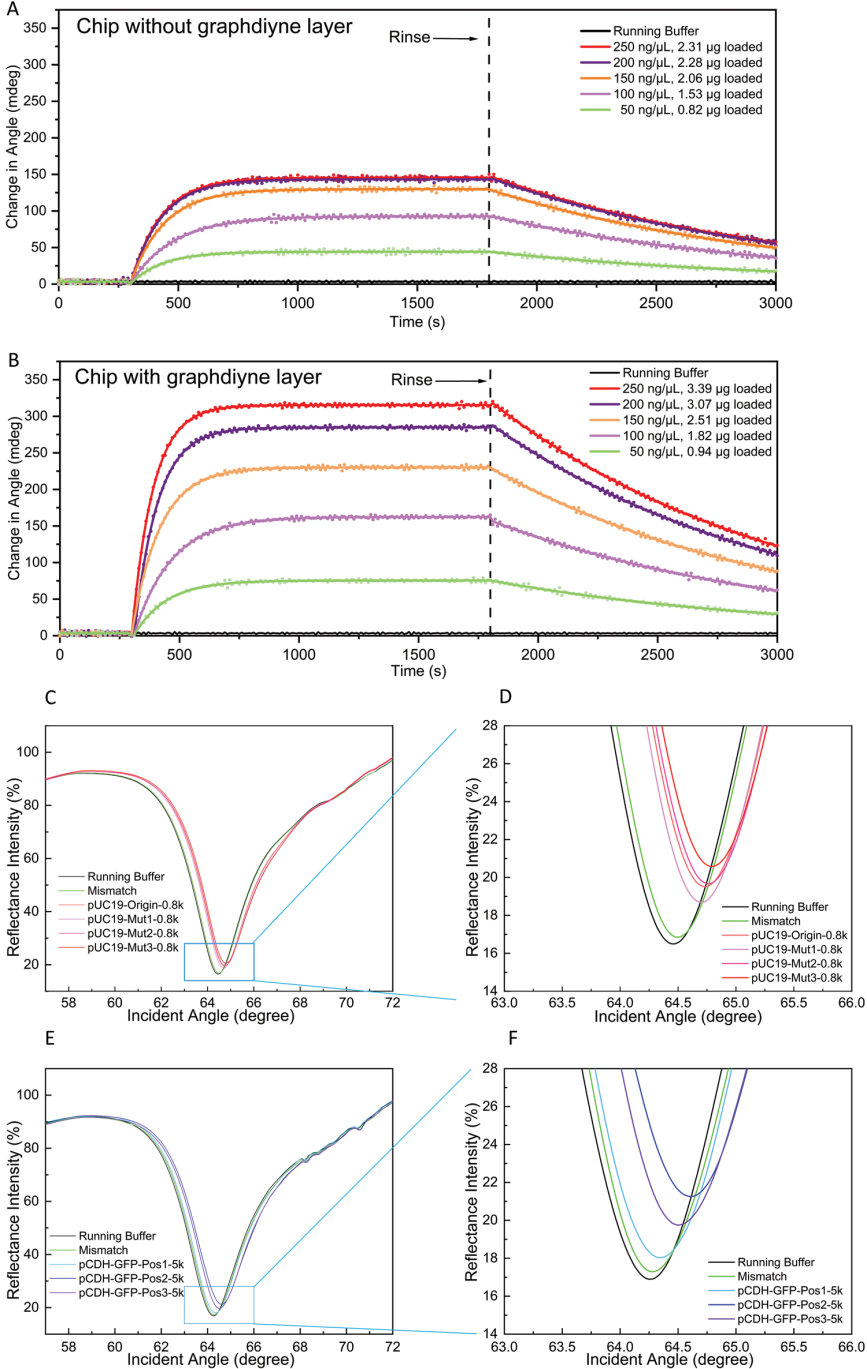
A,B) SPR angular spectra of the dRNP‐functionalized CRISPR‐SPR‐Chips detecting DNA fragments that contain different target sites. C,D) SPR signals generated by CRISPR‐SPR‐Chips with or without a graphdiyne layer immobilized with different loaded amounts of dCas9 protein. E,F) Recombinant pUC19 amplicons (0.8 kb) were detected by the corresponding dRNP‐functionalized CRISPR‐SPR‐Chi(ps (Origin, Mut1, Mut2, and Mut3). G,H) The pCDH amplicons of 5 kb were detected by the dRNP‐functionalized CRISPR‐SPR‐Chips with different target sites (GFP‐Pos1, GFP‐Pos2, and GFP‐Pos3).

Next, the plasmonic performances of the graphdiyne‐modified SPR chip and the unmodified chip were compared by incubating with different concentrations of dCas9 solution, 250 ng µL^−1^ sgRNA, and tested with 1000 ng/µL of pUC19‐MUT3‐0.8k DNA fragments (**Figure** **4**A,B). Importantly, apart from the higher loading capacity, the graphdiyne‐modified SPR chip also exhibited higher sensitivity. Typically, a stronger SPR signal was observed (162.2 mdeg vs. 145.5 mdeg) with a lower loaded amount of dCas9 (Figure 4A,B, 1.82 µg vs. 2.31 µg). Therefore, to achieve the best sensing performances, we ferformed the following experiments only with the graphdiyne‐modified SPR chip and it is referred to as the CRISPR‐SPR‐Chip.

Further, the plasmonic performances of the CRISPR‐SPR‐Chip were systematically analyzed with the samples of recombinant pUC19 amplicons and *GFP*‐carrying pCDH amplicons. The SPR sensing path was adopted to measure the local refractive index changes, displaying the response of an angular reflectance shift. The SPR angular response signal was enhanced by the coupling of the electromagnetic field between the Au film layer and the graphdiyne coating layer. The dRNP molecular system was covalently immobilized to the graphdiyne layer surface via *π*–*π* aromatic stacking. The SPR angular spectra of pUC19‐0.8k and pCDH‐5k are shown as examples in Figure 4C–F (Figure S7, Supporting Information, for pUC19‐0.3k and pCDH‐2k). The pUC19‐0.8k amplicons resulted in an obvious angular shift from 64.45° for running buffer to 64.79° for ‐Mut3 (Figure 4C,D). Similarly, an angular shift for pCDH‐5k samples from 64.25° to 64.62° was observed with the ‐Pos2 target site (Figure 4E,F). Importantly, these SPR angular shifts were consistent with the qPCR results, revealing the specificity and reliability of our SPR detection method. It is worth noting that the SPR sensing response of the refractive index change was mostly caused by the binding of DNA molecules. However, the intrinsic refractive index change of the liquid sample can also lead to a minor angular shift. In our case, the DNA fragments containing non‐target sites (referred to as “mismatch” in figures) can result in a ≈0.01° angular shift, which was further assessed in LOD tests.

**Figure 5:**
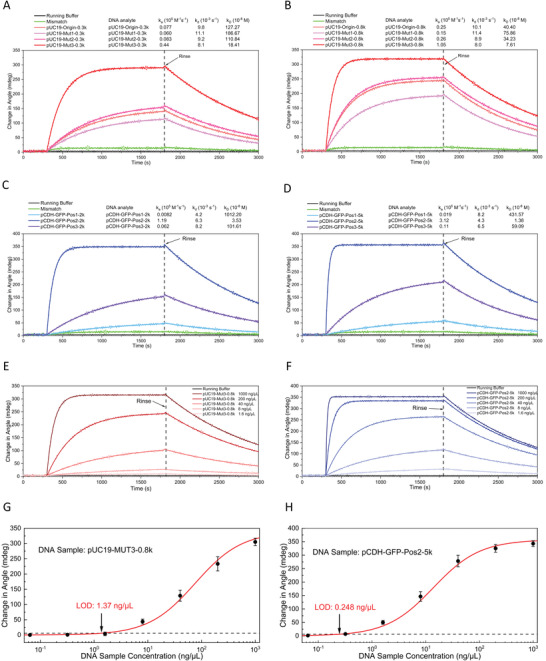
Real‐time SPR response of the dRNP‐functionalized CRISPR‐SPR‐Chips used to monitor the kinetic process of DNA fragments binding to the corresponding dRNP‐functionalized CRISPR‐SPR‐Chips. A, B) Binding kinetics of recombinant pUC19 amplicons (0.3 kb and 0.8 kb). C, D) Binding kinetics of pCDH amplicons (2 kb and 5 kb). (E) Binding kinetics of the pUC19‐Mut3‐0.8k amplicons in concentrations ranging from 1.6 ng µL^−1^ to 1000 ng µL^−1^. (F) Binding kinetics of the pCDH‐GFP‐Pos2‐5k amplicons in concentrations ranging from 1.6 ng µL^−1^ to 1000 ng µL^−1^. G, H) Limit of detection (LOD) test of pUC19‐Mut3‐0.8k and pCDH‐GFP‐Pos2‐5k amplicons (*n*  =  3; error bars represent standard deviations).

An advantage of SPR technology is its ability to accurately quantify the molecular interaction rate, binding level, and kinetic constants.^[^ ^52^ ^]^ We evaluated the binding kinetics of the recombinant pUC19 and pCDH PCR amplicons to their corresponding dRNPs using real‐time SPR monitoring with dRNP‐functionalized chips. In both the ‐0.3k and ‐0.8k groups, the mutagenesis of ‐Mut3 resulted in the fastest association rate, presenting the highest affinitive property (**Figure** **5**A,B). In contrast, the non‐mutated (‐Origin group) amplicons displayed the poorest affinity. Similar results were obtained with the pCDH amplicons, in which the target site of ‐Pos2 exhibited the highest affinity, whereas ‐Pos1 showed the lowest affinity (Figure 5C,D). These results are consistent with the CRISPR‐SPR‐Chip capture efficiency results obtained from qPCR analysis. Moreover, in both pUC19 and pCDH experiments, DNA analytes with a higher molecular weight exhibited better affinity. Next, we chose pUC19‐Mut3‐0.8k and pCDH‐GFP‐Pos2‐5k to perform a series of affinity tests with concentrations ranging from 1.6 ng µL^−1^ to 1000 ng µL^−1^ to determine their kinetic constants.

The plots of the fitted curves for pUC19‐Mut3‐0.8k and for pCDH‐GFP‐Pos2‐5k are shown in Figure 5E,F, respectively and the corresponding kinetics parameters are shown in **Table** **1**. The *K_D_* of pCDH‐GFP‐Pos2‐5k was 5 times higher than that of pUC19‐Mut3‐0.8k, indicating that the CRISPR/dCas molecular capture system exhibited various affinities. The kinetic constants of other DNA amplicons are presented in Figure 5A,B and Table S10, Supporting Information. In addition, the real‐time SPR response could effectively differentiate the target and the mismatched sequences. The signal intensity difference could be observed in the “association period” within 300 s, implying that this sensing time was sufficient for a rapid genomic analysis. Therefore, the analysis time required for CRISPR‐SPR‐Chip can be considered as ≈5 min.

**Table 1:** Kinetic constants for the interaction of the CRISPR‐SPR‐Chip and DNA analytes

DNA analyte	*k*_a_ [10^5^ M^−1^s^−1^]	*k*_d_ [10^−3^ s^−1^]	*K*_D_ [10^−8^ M]
pUC19‐Mut3‐0.8k	1.05	8.0	7.61
pCDH‐GFP‐Pos2‐5k	3.12	4.3	1.38

To evaluate the limit of the sensing performance of the CRISPR‐SPR‐Chip, pUC19‐Mut3‐0.8k and pCDH‐GFP‐Pos2‐5k were measured over the concentration range of 64 pg µL^−1^ to 1000 ng µL^−1^. The CRISPR sensing system started to attain the saturation condition when the concentration of DNA analytes reached 1000 ng µL^−1^ (Figure 5G–H). In contrast, a low concentration of 64 pg µL^−1^ only resulted in a weak SPR response, which was similar to that of the system blank. According to IUPAC guidelines,^[^ ^53^ ^]^ the LOD of the CRISPR sensing system is estimated as the sum of the blank measures (running buffer) plus triple its standard deviation (i.e., 3.41 + 3 × 0.87 = 6.02 mdeg). By fitting with the calibrated regression curve, the LOD of pUC19‐Mut3‐0.8k and pCDH‐GFP‐Pos2‐5k was calculated to be 1.37 ng µL^−1^ and 0.248 ng µL^−1^, respectively. Thus, the detectable DNA fragment concentration corresponding to the systematic LOD was about 1.3 fM (≈1.57 × 10^3^ copies in 2 µL analyte solution), which is higher than that reported in a previous study.^[^ ^20^ ^]^

### 2.9 Detection of DMD Clinical Specimens using the CRISPR‐SPR‐Chip

**Figure 6:**
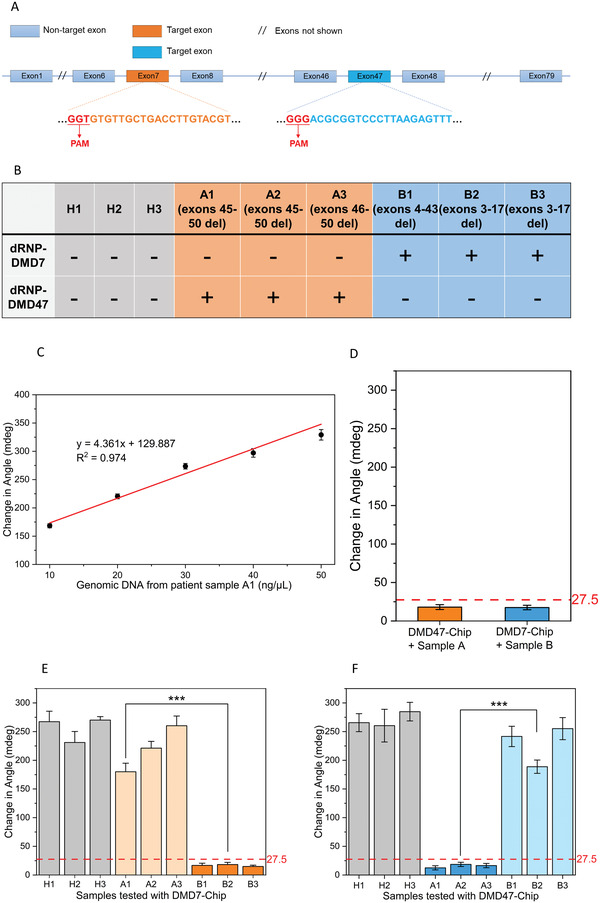
Analysis of healthy and DMD clinical samples for DMD‐associated dystrophin exon 7 and exon 47 deletions using CRISPR‐SPR‐Chips. A) Schematic of the dystrophin gene (DMD) with target exons. B) Results of CRISPR‐SPR‐Chip analysis for the deletion (absence) of targeted exons (+) in healthy and DMD clinical samples. C) Change in angle obtained from the SPR measurement of the dRNP‐DMD7‐CRISPR‐SPR‐Chip with patient sample A1 using gradient concentrations. D) Negative signal threshold of CRISPR‐SPR‐Chips. E) Change in angle obtained from the SPR measurement of the dRNP‐DMD7‐CRISPR‐SPR‐Chip with all samples. The negative signal threshold is the result from panel D. F) Change in angle obtained from the SPR measurement of the dRNP‐DMD47‐CRISPR‐SPR‐Chip with all samples. The negative signal threshold is the result from panel D. All data were collected from three independent experiments (***p < 0.001, two‐tailed *t*‐test, *n*  =  3; error bars represent standard deviations).

The ultimate purpose of this study was to develop a CRISPR‐SPR methodology to detect gene mutations in clinical specimens from patients with suspected DMD. One reason for focusing on this disease is that it is typically associated with large deletions in exons 2–10 and 45–55 of DMD,^[^ ^44^ ^]^ and such large deletions are easy to detect using CRISPR‐SPR‐Chips targeting two sequences in exon 7 and exon 47. Moreover, CRISPR‐SPR‐Chips can provide fast and convenient gene mutation screening to facilitate recently emerging genome editing therapies for DMD at an early age.^[^ ^42^ ^]^ In this experiment, two types of CRISPR‐SPR‐Chips specific to exon 7 and exon 47 of DMD (**Figure** **6**A, highlighted) were applied for detection in clinical samples.

The correlation between sample concentration and SPR response was evaluated using the dRNP‐DMD7‐CRISPR‐SPR‐Chip and sample A1 (exon 7 present) in concentrations ranging from 10 to 50 ng µL^−1^. Within this range, the change in angle and DNA sample concentrations showed favorable linearity, indicating that the output signal in the following experiments was steady and reliable (Figure 6C). Moreover, the sensitivity down to 10 ng µL^−1^ confirmed the amplification‐free detection feature of the CRISPR‐SPR‐Chip, in which the minimum required quantity of genomic material is comparable to that obtainable from non‐invasive commercial buccal sampling methods.^[^ ^20^ ^]^

The positive threshold for defining “DMD illness” was then determined using DNA samples (1000 ng µL^−1^) with the corresponding CRISPR‐SPR‐Chips. Samples A1–A3 with deletion of exon 47 were detected by the dRNP‐DMD47‐CRISPR‐SPR‐Chip and Samples B1–B3 with deletion of exon 7 were detected by the dRNP‐DMD7‐CRISPR‐SPR‐Chip. As shown in Figure 6D, only weak SPR responses (angle change) were observed due to the intrinsic refractive index change of the liquid environments. Therefore, a “positive” threshold was defined as the average of the “positive” measures plus triple its standard deviation. As a result, the “positive” threshold for DMD47 and DMD7 was calculated as 27.48 mdeg [18.03 + (3.75 × 3)] and 26.29 mdeg [17.50 + (2.93 × 3)], respectively. To prevent possible false results, we set the universal positive threshold as 27.5 mdeg. Specifically, DNA from clinical samples lacking the target exons, even at the highest concentration (1000 ng µL^−1^), in our CRISPR‐SPR sensing experiment cannot result in an angle change of more than 27.5 mdeg. Samples with an angle change below this threshold indicate absence of the target exons, and can therefore be interpreted as “positive” and considered as “DMD illness,” and vice versa.

Based on the defined positive threshold, all clinical samples were tested for the absence of exon 7 and exon 47 absence detection using their corresponding CRISPR‐SPR‐Chip. DNA samples from healthy individuals (H1, H2, and H3) exhibited an angle change greater than 200 mdeg when tested with both ‐DMD7 and ‐DMD47 CRISPR‐SPR‐Chips (Figure 6E–F), which was substantially larger than the positive threshold of 27.5 mdeg, confirming the presence of both exons 7 and 47 in healthy samples. In contrast, patient samples with exon deletions showed a significant reduction in the signal output when exposed to their corresponding CRISPR‐SPR‐Chips. Patient samples A1, A2, and A3 presented a change in angle of 12.5, 18.5, and 16.4 mdeg, respectively, when tested with the dRNP‐DMD47‐CRISPR‐SPR‐Chip, while samples B1, B2, and B3 showed a change of 16.8, 18.3, and 15.0 mdeg, respectively, with the dRNP‐DMD7‐CRISPR‐SPR‐Chip. These notably weak SPR responses indicated the absence of exon 47 and exon 7 in groups A and B, respectively. Importantly, all patient samples showed SPR responses that were comparable to those of healthy samples when tested with the “mismatch” CRISPR‐SPR‐Chip, suggesting that these patient genomic samples contained the counterpart exons.

These results provide strong validation that CRISPR‐SPR‐Chips can accurately detect the absence of exons 7 and 47 in an unamplified human genomic sample, thereby identifying the gene deletion feature of patients with DMD. Although only two target exons were used as a demonstration example in our clinical sensing tests, these results demonstrate that the CRISPR‐SPR‐Chip possesses expandable potential to detect any target gene for multiple purposes by simply replacing the specific sgRNA sequences. To more comprehensively assess the sensing performance, we benchmarked the existing CRIPSR‐based biosensors in comparison with other nucleic acid detection platforms (see Table S11, Supporting Information). Overall, the novel CRISPR‐SPR‐Chip developed in this work exhibited a competitive performance in terms of both the LOD and detection time.

## 3 Conclusions 

A label‐free and rapid SPR biosensor integrated with 2D graphdiyne and the CRISPR molecular system was developed for detecting a target sequence in genomic DNA. Benefiting from the ability of the anchored dRNPs to rapidly and accurately scan the genome, the novel CRISPR‐SPR‐Chip exhibited outstanding sensing performance with high sensitivity, specificity, and repeatability. The CRISPR‐SPR‐Chip could distinguish single‐site mutations introduced in a gene when sensing with the experimentally designed PCR amplicons. The locally grown graphdiyne film endows advantageous features of the high loading capacity of dCas9 nuclease and signal enhancement via electromagnetic field coupling, allowing the SPR sensing platform to achieve detection of the target DNA sequence with an LOD of 1.3 fM. The clinical utility of the CRISPR‐SPR‐Chip was evaluated for sensing exon 7 and exon 47 genetic deletions in patients with DMD without any pre‐amplification step. By simply altering the unique 20‐nucleotide sgRNA sequence, the CRISPR‐SPR‐Chip successfully detected various genetic mutations. The CRISPR‐SPR‐Chip has the potential to not only improve access to genetic information with hereditary disease diagnosis, but its real‐time monitoring can also be applied as a powerful tool for investigation of the affinity kinetics of sgRNAs to various DNA sequences. Nevertheless, the relatively low reusability of the sensing system could be considered a limitation, since the regeneration buffer used for the conventional metal‐based SPR chip could undermine the structural integrity of the as‐grown graphdiyne film. The ready‐prepared sensor chips are fixed with the pre‐designed sgRNA sequences, which may increase their usage cost and limit the feasibility of analyzing multiple gene sequences of interest from a single genomic sample. In future studies, this system could be improved by functionalizing with other 2D materials to further improve the SPR signal response; integrating with microfluidic technology for multiplex gene detection; and replacing the dCas9 nuclease with other Cas proteins, such as Cas 13a (for detection of RNA‐based sequences) or Cas 12a (for sensing single‐stranded DNA), which might significantly increase the application potential of the CRISPR‐SPR‐Chip in various fields of experimental and clinical research. With the integrated sequence‐specific recognizing ability of the CRISPR/Cas system and the high sensitivity of the SPR sensor, we provide a novel CRISPR empowered SPR (CRISPR‐eSPR) sensor for precise, stable, sensitive, and reliable gene analysis of clinical samples.

## 4 Experimental Section

### Preparation of Graphdiyne Film on SPR Chips

Graphdiyne was prepared according to a previously reported protocol^[^ ^54^ ^]^ with minor modifications. The detailed preparation methods and characterization of the film are provided in the Supplementary Methods: Preparation of graphdiyne film on SPR chips, and Material characterization.

### sgRNA Design

All of the sgRNAs targeting recombinant pUC19 [New England Biolabs (NEB), Ipswich, MA, USA] vectors (Origin, Mut1, Mut2, and Mut3), the green fluorescent protein (GFP) sequence in the pCDH (NEB) vector (GFP‐Pos1, GFP‐Pos2, and GFP‐Pos3), and exons 7 and 47 of human DMD were designed using DNA 2.0 (now ATUM) CRISPR DNA design tool. The sgRNAs were designed in accordance with the mutated and unmutated sequences, and used to form CRISPR‐sgRNA complexes (RNPs) with Cas9 nuclease (*S. pyogenes*; NEB) are referred to as RNP‐Origin, RNP‐Mut1, RNP‐Mut2, and RNP‐Mut3. All sgRNA sequences are presented in Supplementary Tables S1–S3, Supporting Information. High‐performance liquid chromatography‐purified sgRNAs were obtained from Thermo Fisher Scientific (Waltham, MA, USA).

### Site‐Directed Mutagenesis

To evaluate the specificity of the RNPs for their target double‐stranded DNA (dsDNA), we constructed various DNA sequence targets by site‐directed mutagenesis. A wild‐type pUC19 vector (2686 bp, Figure S1A, Supporting Information) was selected as a blank DNA template, and three types of mutations (three continuous bases, six continuous bases, and every three bases) were introduced to the *lacZα* gene region. Specifically, the mutated sites were derived from the wild‐type pUC19 (gtcgact) using recombinant gene sequences Mut1 (gtcgCAC), Mut2 (gGTTCAC), and Mut3 (CtcCacA) (Figure S2, Supporting Information), which were constructed using GENEART Site‐Directed Mutagenesis System (Thermo Fisher Scientific) with three primer pairs (Table S4, Supporting Information). The detailed mutagenesis procedures are described in the Supplementary Methods: Site‐directed mutagenesis.

### Preparation of DNA Fragments Containing Different Target Sequences

Since sgRNAs exhibit different binding efficiencies^[^ ^55^ ^]^ to target genes, we utilized PCR amplicons of a pCDH vector (8205 bp, Figure S1B, Supporting Information) containing the *GFP* gene, which is widely applied for validating CRISPR‐Cas gene editing,^[^ ^13^, ^56^ ^]^ to estimate the binding efficiency of the RNP. The DNA fragments with different target sequences were prepared by PCR with the pUC19 plasmid mutagens (Origin, Mut1, Mut2, and Mut3) and pCDH vector (GFP‐Pos1, GFP‐Pos2, and GFP‐Pos3). Primers for each target sequence were designed using Primer Premier 6.0 (Tables S5–S8, Supporting Information). The 2125 bp amplicon is referred to as pCDH‐GFP‐2k, and its two‐sided extended 4994 bp DNA fragment is referred to as pCDH‐GFP‐5k. As a negative control, two DNA fragments without *GFP* (2075 bp and 4806 bp, respectively) were obtained and named pCDH‐noneGFP‐2k and pCDH‐noneGFP‐5k. Three sgRNAs (Table S8, Supporting Information) targeting different positions (Figures S3 and S4, Supporting Information) of *GFP* were designed and their RNPs with Cas9 nuclease are referred to as RNP‐GFP‐Pos1, RNP‐GFP‐Pos2, and RNP‐GFP‐Pos3. The fragments were confirmed and prepared by PCR (see, Supporting Information).

### In Vitro Cleavage Assay

The DNA fragments containing different target sites were cleaved in vitro with their corresponding Cas9‐sgRNA complexes. Each sample (400 ng) was mixed with 1 µg of Cas9 protein, 1 µg of sgRNA, 3 µl of NEBuffer 3.1 (NEB), and nuclease‐free water into a 30 µL reaction mixture. After pre‐incubation at 25 °C for 5 min, the mixtures were incubated for 1 h at 37 °C. The reaction was terminated by heating at 85 °C for 10 min, followed by adding 1 µL of proteinase K (NEB) and incubating at 60 °C for 10 min to remove the Cas9 protein. Finally, the samples were diluted and analyzed using agarose gel electrophoresis (see Supporting Information).

### In Vitro Capture Assay of Target DNA Fragments

The in vitro capture assay method was the same as that for the in vitro cleavage assay except that Cas9 protein was replaced with dCas9 protein (6 × His‐tag, NEB). After incubation at 37 °C for 1 h, the samples were treated as follows: no subsequent treatment (control), heating at 85 °C for 10 min, and incubating with 1 µL of proteinase K at 60 °C for 10 min. The samples were analyzed using agarose gel electrophoresis (see detailed illustration in Supporting Information).

### In Vitro Binding Assay

The in vitro binding assay was performed with DNA fragments using magnetic beads (MBs) to evaluate the affinity of dCas9‐sgRNA (dRNP) to the target sequences (Figure 3F). After magnetic separation, the uncaptured amplicons that remained in the supernatant were further analyzed using quantitative PCR to determine the capture efficiency and the relative affinity of the immobilized dRNPs to their target DNA sequences (see detailed illustration in Supplementary Methods: Magnetic bead separation, electrophoresis, and qPCR analysis).

### Immobilization of dCas9‐sgRNA on the Graphdiyne Film Surface

The fabricated graphdiyne‐deposited SPR chip was washed twice with ethanol and ultrapure water and then functionalized with 1‐pyrenebutyric acid N‐hydroxysuccinimide ester (PBASE; Sigma‐Aldrich, St. Louis, MO, USA), which served as a linker molecule to provide activated N‐hydroxysuccinimide ester (NHS) groups on the surface of graphdiyne via *π–π* interactions.^[^ ^57^ ^]^ The graphdiyne‐deposited SPR chips were immersed in a 25 mM PBASE solution in dimethylformamide (Aladdin) for 1 h at room temperature, washed three times with ethanol and ultrapure water, and dried under purging N_2_. Subsequently, 50–400 ng µL^−1^ (20 µL in PBS buffer, pH 7.4) of dCas9 protein was dropped onto the surface of the chip, which was then incubated for 1 h at room temperature, followed by rinsing with ultrapure water to remove unreacted protein. After collecting all of the protein residue, the protein concentration was measured using the bicinchoninic acid (BCA) assay to determine the amount of protein to be loaded on the chips.

Furthermore, the unreacted PBASE molecules were blocked by incubating with 10 mM ethanolamine aqueous solution (pH 8.5) for 30 min. After blocking, 50–400 ng µL^−1^ (20 µL in 2 mM MgCl_2_) of sgRNA specific to the target sequence was introduced onto the graphdiyne film surface, which was then incubated at 37 °C for 15 min to form the dCas9‐sgRNA complex. Finally, the chip was washed with 2 mM MgCl_2_ for 5 min to remove any unbound sgRNA, the RNA loading amount was calculated using a method similar to that used to measure protein: the RNA residue was measured using a NanoDrop spectrophotometer (Thermofisher Scientific). The immobilization of dCas9‐sgRNA complex was proved (see Supporting Information: Verification of the immobilization of dCas9 and sgRNA). Finally, the fully functional CRISPR‐SPR‐Chip was obtained.

### SPR Measurement

For the SPR measurement, the plasmonic performances of the graphdiyne‐modified SPR sensor and SPR sensors without graphdiyne were first compared after incubating with different concentrations of dCas9 protein, 250 ng µL^−1^ sgRNA specific to pUC19‐MUT3, and 1000 ng µL^−1^ of pUC19‐MUT3‐0.8k fragment. The graphdiyne‐modified SPR sensor performed better than the SPR sensors without graphdiyne; therefore, it was further systematically analyzed with all the samples of recombinant pUC19 amplicons and *GFP*‐carrying pCDH amplicons. In our SPR instrument, an attenuated total reflection (ATR) setup was used for the excitation of surface plasmons using the Kretschmann configuration. A ZK7 semi‐cylindrical prism was applied with a collimated p‐polarized light‐emitting diode (LED; *λ* = 640 nm), which emits incident light into the interface of the SPR chip at an inclined nominal incident angle of 72°. A high‐speed luminance spectrometer was used to measure the reflectance intensity that corresponded to the ATR light from the center of the SPR chip. The ready‐prepared CRISPR‐SPR‐Chip was clamped with the prism against a Teflon sample chamber, which served as a mini‐incubator throughout the SPR test. The prism‐chamber setup was mounted on a two‐axis goniometer to measure the SPR angle.

In a typical SPR test, 2 µL of DNA sample (2 mM MgCl_2_) with the desired concentration was introduced into the Teflon sample chamber via a capillary tube over 30 min at 37 °C. After incubation, the CRISPR‐SPR‐Chip was rinsed with 20 µL of 2 mM MgCl_2_ for 20 min. The SPR response was monitored in real time for further analysis. All of the SPR curves were smoothed using the Savitzky‐Golay method. The calculations for investigating the binding kinetics among the varied sites are provided in the Supplementary Methods: Binding kinetics parameters.

### Detection of DMD‐Associated Clinical Specimens

Human genomic samples from healthy controls and patients with DMD were obtained with a certificate of analysis from the Coriell Institute for Medical Research (Camden, NJ, USA). Samples of patients with DMD were tested for detection of exon 47 deletion in DMD (A1: NA05017, exon 45–50 deletion; A2: NA05016, exon 45–50 deletion; and A3: NA03929, exon 46–50 deletion) or exon 7 deletion (B1: NA05170, exons 4–43 deletion; B2: NA03780, exons 3–17 deletion; B3: NA03782, exons 3–17 deletion). Healthy samples H1 (NA03349), H2 (NA03798), and H3 (NA17508) were identified for the presence of both exon 47 and exon 7 (Table S9, Supporting Information).

Since the DMD47‐CRISPR‐SPR‐Chip was designed to detect the deletion of exon 47, samples A1, A2, and A3 (exon 47 deletion) should theoretically show “positive” signals (i.e., an SPR response below a certain value). The same method was implemented for the DMD7‐CRISPR‐SPR‐Chip. The threshold was defined based on the International Union of Pure and Applied Chemistry (IUPAC) calibration method: average of the “positive” measures plus triple its standard deviation.

The CRISPR‐SPR‐Chips (DMD7‐ and DMD47‐) were incubated with all nine patient samples, and SPR data were collected and analyzed. “Positive” or “negative” signals of each sample were identified according to our defined threshold.

### Statistical Analysis

All measurements were performed in triplicate (*n* = 3), and the data are displayed as mean ± standard deviation. Correlations were performed with linear regression to determine the goodness of fit (Pearson's correlation coefficient, *R* ^2^). For inter‐sample comparisons, multiple pairs of samples were analyzed by a two‐tailed *t*‐test, and the resulting *p* values were adjusted for multiple hypothesis testing using Bonferroni correction. *p < 0.05, **p < 0.01, and ***p < 0.001 indicate obvious statistical differences. All statistical analyses were performed using OriginPro (v.2019b).

## Conflict of Interest

The authors declare no conflict of interest.

## Author Contributions

F.Z. and Z.C. contributed equally to this work and are co‐first authors. F.Z.: Conceptualization, Methodology, Formal analysis, Investigation, Data Curation, and Writing. Z.C.: Conceptualization, Software, Validation, Investigation, Writing and Visualization. J.L.: Investigation. R.W.: Data Curation. B.Z.: Resources. G.N.: Resources. Z.X.: Funding acquisition. H.Z.: Supervision, Project administration, and Funding acquisition.

## Supporting information

Supporting Information

## Data Availability

The data that support the findings of this study are available from the corresponding author upon reasonable request.
