# Supplementary material for: A Highly Sensitive CRISPR‐Empowered Surface Plasmon Resonance Sensor for Diagnosis of Inherited Diseases with Femtomolar‐Level Real‐Time Quantification
Source: Adv Sci (Weinh). 2022 Mar 27;9(14):2105231. doi: 10.1002/advs.202105231 (PMC9108660; doi:10.1002/advs.202105231)
Supplement: Supplementary file 1 — Supporting Information [file ADVS-9-2105231-s001.pdf]

ADVANCED SCIENCE Open Access

Supporting Information

for *Adv. Sci.*, DOI 10.1002/advs.202105231

A Highly Sensitive CRISPR-Empowered Surface Plasmon Resonance Sensor for Diagnosis of Inherited Diseases with Femtomolar-Level Real-Time Quantification

Fei Zheng, Zhi Chen, Jingfeng Li, Rui Wu, Bin Zhang, Guohui Nie, Zhongjian Xie and Han Zhang**

A Highly Sensitive CRISPR-empowered Surface Plasmon Resonance Sensor for Diagnosis of Inherited Diseases with Femtomolar-level Real-time Quantification

Supplementary Information

Fei Zheng [1,#], Zhi Chen [1,#], Rui Wu [2], Bin Zhang [1], Guohui Nie [1], Zhongjian Xie [3,4,*], Han Zhang [1,*]

[1] Shenzhen Engineering Laboratory of phosphorene and Optoelectronics; International Collaborative Laboratory of 2D Materials for Optoelectronics Science and Technology of Ministry of Education, Shenzhen Institute of Translational Medicine, Department of Otolaryngology, Shenzhen Second People's Hospital, the First Affiliated Hospital, Institute of Microscale Optoelectronics, Shenzhen University, Shenzhen, 518060, P.R. China

[2] Laboratory of Robotics and System, Harbin Institute of Technology, Harbin, 150001, P. R. China

[3] Institute of Pediatrics, Shenzhen Children's Hospital, Shenzhen 518038, Guangdong, P. R. China

[4] Shenzhen International Institute for Biomedical Research, Shenzhen 518116, Guangdong, P. R. China

[#] These authors contributed equally to this work

[*] Corresponding author: Zhongjian Xie (email: zjxie2011@163.com) and Han Zhang (email: hzhang@szu.edu.cn)

Appendix A. Supplementary Methods

Preparation of graphdiyne film on SPR chips

A CVD- grade copper foil was trimmed into 20 × 20 mm^2 pieces, which were sonicated with hydrochloric acid (4 M, 100 mL) for 3 min to remove the surface copper oxides. The copper pieces were then washed with ultrapure water and ethanol with sonication, followed by drying under purging N_2. The cleaned copper pieces and the bare SPR chips (bare Au coating) were alternately placed on a steel-made glass slide rack at 1 mm intervals; immersed into a mixed solution of N,N,N',N'-tetramethylethylenediamine (1 mL), pyridine (5 mL), and ethyl acetate (50 mL) (all from Aladdin Co., Ltd., Shanghai, China); and heated to 60°C for 2 h with continuously bubbling argon. In an alkaline solution, copper can transfer to Cu-N ligand complexes that act as a "running catalyst," diffusing from the copper foil to the SPR chip surfaces for the local acetylenic coupling reaction. The monomer precursor of graphdiyne, hexakis[(trimethylsilyl)ethynyl] benzene (Xianfeng Nano Co., Ltd., Nanjing, China), with a mass of 25 mg, was treated by reacting with tetra-*n*-butylammonium fluoride (20 mM; Aladdin) in 50 mL of tetrahydrofuran (Aladdin) under an argon atmosphere. The reaction was carried out with continuous stirring in an ice bath for 20 min, obtaining a light purple-colored solution. The resulted solution was then diluted with 50 mL of ethyl acetate and washed in a saturated NaCl aqueous solution three times using a separatory funnel. The organic solution was dried with anhydrous $MgSO_4$, filtered, and dried under a vacuum at 30°C in the dark. The obtained yellowish product was redissolved in 50 mL of ethyl acetate

and then added dropwise into the above-mixed solution for 6 h. The reaction system was maintained at 60°C for 48 h. Upon completing the acetylenic coupling reaction, homogenous graphdiyne films formed on the surface of SPR chips, which were washed with acetone and ethanol, dried with N_2, and stored in the dark until further use. The graphdiyne flakes suspended in the reaction solution were also collected, washed, and vacuum-dried for further characterization.

Site-directed mutagenesis

First, 1.5 μL of primers (10 μM), 1 μL of plasmid DNA (20 ng/μL), 13.4 μL of the reaction mix, and 34.1 μL nuclease-free water were mixed well and incubated at 37°C for 12 min to complete the DNA methylation reaction. Mutagenesis PCR was then performed with pre-denaturation (94°C for 2 min); 18 thermal cycles of denaturation at 94°C for 20 s, annealing at 57°C for 30 s, and extension at 68°C for 1 min; and final extension at 68°C for 5 min. To enhance the mutagenesis efficiency, 4 μL of the resulted product was incubated with 16 μL of the enzyme mix at room temperature for 10 min to remove unmethylated templates. The reaction was terminated with the addition of a 1 μL ethylenediaminetetraacetic acid solution (0.5 M), and the recombinant plasmid DNA was obtained for the transformation process.

In a typical transformation procedure, a vial of 50 μL DH5-T1 competent cells (Thermo Fisher Scientific, Waltham, MA, USA) was thawed on ice within 20 min and 2 μL of the recombinant plasmid DNA was gently pipetted into the vial. The mixture was

incubated on ice for 12 min, followed by incubating in a water bath for exactly 30 s at 42°C. Each vial of cells was cooled down on ice for 2 min and incubated with 250 µL of pre-warmed SOC medium (Thermo Fisher Scientific) at 37°C for 1 h in a shaking incubator (225 rpm). The transformed cells were diluted by 20 times with SOC medium and 100 µL of the diluted cell suspension was inoculated to a Luria-Bertani–ampicillin plate using a steel-made scraper. The inoculated plates were inverted and incubated at 37°C for 16–20 h. Three colonies on each plate were picked up for further fermentation and plasmid isolation.

Material characterization

Low-resolution transmission electronic microscopy and high-resolution transmission electron microscopy images were captured by a JEM 2100 (JEOL, Japan) and FEI Tecnai TF30 (Thermo Fisher Scientific, Waltham, MA, USA) microscope, respectively. Nanoscale morphology was observed using atomic force microscopy (Bruker Dimension Ico). The X-ray photoelectron spectroscopy data were collected using an ESCALAB 250Xi XPS spectrometer (Thermo Fisher Scientific). Polarized Raman spectroscopy was carried out using an iHR 320 spectrometer (Horibai, Japan).

The concentration of all DNA samples was determined with Nanodrop UV-Vis spectrophotometers (Thermo Fisher Scientific). The concentration of all protein samples was measured using a BCA assay kit (Thermo Fisher Scientific) with an Epoch microplate reader (BioTek, USA).

Magnetic bead separation, electrophoresis, and qPCR analysis

Carboxylated magnetic beads (MBs; 1 μm, Dynabeads MyOne Carboxylic Acid, Thermo Fisher Scientific) were activated by incubating with 10 mM 1-ethyl-3-(3-dimethylaminopropyl) carbodiimide hydrochloride (Aladdin) and 20 mM sulfo-N-hydroxysulfosuccinimide sodium salt (sulfo-NHS; Aladdin) in 0.1 M 2-(N-morpholino)ethanesulfonic acid buffer (pH 6.0; Thermo Fisher) containing 0.5 M NaCl (pH 6.0) for 15 min at room temperature. The activated MBs were separated and promptly coupled with dCas9 protein (250 ng/μL) in phosphate-buffered saline (PBS, pH 7.4; Thermo Fisher) for 4 h in a cross-linking reaction. After dCas9 coupling, the MBs were blocked with 1.0 M glycine solution (pH 7.4) for 30 min at room temperature. The dCas9-coupled MBs were incubated with sgRNA (250 ng/μL) in a 2 mM $MgCl_2$ solution for 30 min at 37°C to form fully functional CRISPR-MBs. The binding assay was carried out by introducing DNA samples into the CRISPR-MBs suspension (in 2 mM $MgCl_2$) and incubated for 1 h at 37°C. Agarose gel electrophoresis was performed with 0.8–2% agarose gel in TAE buffer (pH 8.3), depending on the lengths of DNA samples. The samples were pre-mixed with ethidium bromide (1:10000, v:v) and run under 105 V for 30 min. Gel images were visualized using an ultraviolet transilluminator (Bio-Rad, USA).

The qPCR assay was performed using KOD SYBR qPCR Mix kit (Toyobo, Japan) with StepOne™ Plus Real-Time PCR System (Applied Biosystems, Foster City, CA, USA). The reaction system contained KOD SYBR qPCR Mix, 0.2 μM of each

primer (forward and reverse), 1 ng of diluted DNA, and DNase-free water up to 20 μL. Thermal cycling was carried out for 2 min at 98°C for initial denaturation, followed by 30 cycles of denaturation at 98°C for 10 s, annealing at 60°C for 10 s, and extension at 68°C for 30 s. The melting curve was recorded from 60°C to 95°C. As a negative control, PCR amplicons were incubated with the empty dCas9-functionalized MBs (without sgRNA), and the cycle threshold (Ct) value of the supernatant was used as the quantification reference.

Furthermore, capture efficiency was calculated by the *Ct* value according to the following equation (1):

$$Capture\ Efficiency\ (\%) = \left(1 - 2^{-(Ct_{sample} - Ct_{reference})}\right) \times 100\% \qquad (1)$$

where Ct_{sample} is the *Ct* value of the target DNA sample that remained in the supernatant after incubating with CRISPR-MBs (colored in amplification curves), and $Ct_{reference}$ is the *Ct* value obtained from the negative control (black in amplification curves).

The DNA fragments with different target sequences were prepared by PCR with the pUC19 plasmid mutagens (Origin, Mut1, Mut2, and Mut3) and pCDH vector (GFP-Pos1, GFP-Pos2, and GFP-Pos3). Primers for each target sequence were designed by Primer Premier 6.0 (Tables S5–S8). PCR was performed using Taq PCR Master Mix Kit (QIAGEN, Hilden, Germany). The reaction system contained PCR buffer, 2.5 units of Taq DNA Polymerase, 200 μM of each dNTP, 0.2 μM of each primer (forward and reverse), 1 ng diluted DNA, and DNase-free water up to 50 μL. PCR was performed using a gradient PCR cycler (Eppendorf, Hamburg, Germany). Thermal

cycling was carried out as follows: 3 min at 94°C for initial denaturation, followed by 30 cycles of denaturation at 94°C for 30 s, annealing at 52–62°C (according to the melting temperature of each primer pair) for 30 s, and extension at 72°C for 1–5 min (depending on the product length).

Verification of the immobilization of dCas9 and sgRNA

The chip-immobilized dcas9 protein was quantified using a modified method based on a similar principle with ELISA. After the immobilization using different concentrations of dCas9 protein solution on the graphdiyne chips, the chips were rinsed and trimmed into a proper size and put into a 96-well plate. After blocking with Blocking One buffer (Nacalai Tesque, Inc.) for 2 h, the chips were incubated with a rabbit monoclonal anti-CRISPR-Cas9 antibody (Abcam, ab189380) for overnight at 4°C, followed by rinsing and incubating with a horseradish peroxidase (HRP)-conjugated goat anti-rabbit IgG H&L secondary antibody (Abcam, ab6721) for 2 h at room temperature. Rinsed, and incubated with a luminol-based enhanced chemiluminescent substrate (SuperSignal™ West Femto Maximum Sensitivity Substrate, Thermofisher Scientific, 34095). Finally, the absorbance at 450 nm of each well was read by using a microplate spectrophotometer (Epoch).

The anchored sgRNA was quantified following a enzymatic release using proteinase K. After the immobilization of dCas9 and different concentrations of sgRNA, the chips were rinsed and incubated with 1 μL proteinase K in 20 μL rinsing buffer at 60 °C

under gently shaking for 10 min. Also, a chip incubated without proteinase K was used for comparison, and all the buffer was collected for the following analysis. Agarose gel electrophoresis was performed with 2.5% agarose gel in TAE buffer (pH 8.3). The samples were pre-mixed with ethidium bromide (1:10000, v:v) and run under 55 V for 90 min at 4 °C. Gel images were visualized using an ultraviolet transilluminator (Bio-Rad, USA).

Binding kinetics parameters

To investigate the difference of binding kinetics among the varied target sites, the concentration of the pUC19 and pCDH amplicons was maintained at 1000 ng/μL. The molecular kinetic interactions can be described as the following equation:

$$\frac{dC_{PA}}{dt} = k_a C_P C_A - k_d C_{PA} \qquad (2)$$

where k_a is the association rate constant; k_d is the dissociation rate; and C_p, C_A, and C_{PA} represent the concentration of the probe, analyte, and their molecular complex, respectively. By introducing the relative SPR response change R, equation (2) can be rewritten as:

$$\frac{dR}{dt} = k_a R_{max} C_A - (k_d + k_a C_A) R \qquad (3)$$

where the maximum SPR response R_{max} can be inferred by the results for the DNA analyte at 1000 ng/μL when CRISPR-SPR-Chip reached saturation. The equilibrium constant K_D is determined using equation (4):

$K_D = k_d / k_a$ (4).

Appendix B. Supplementary Figures

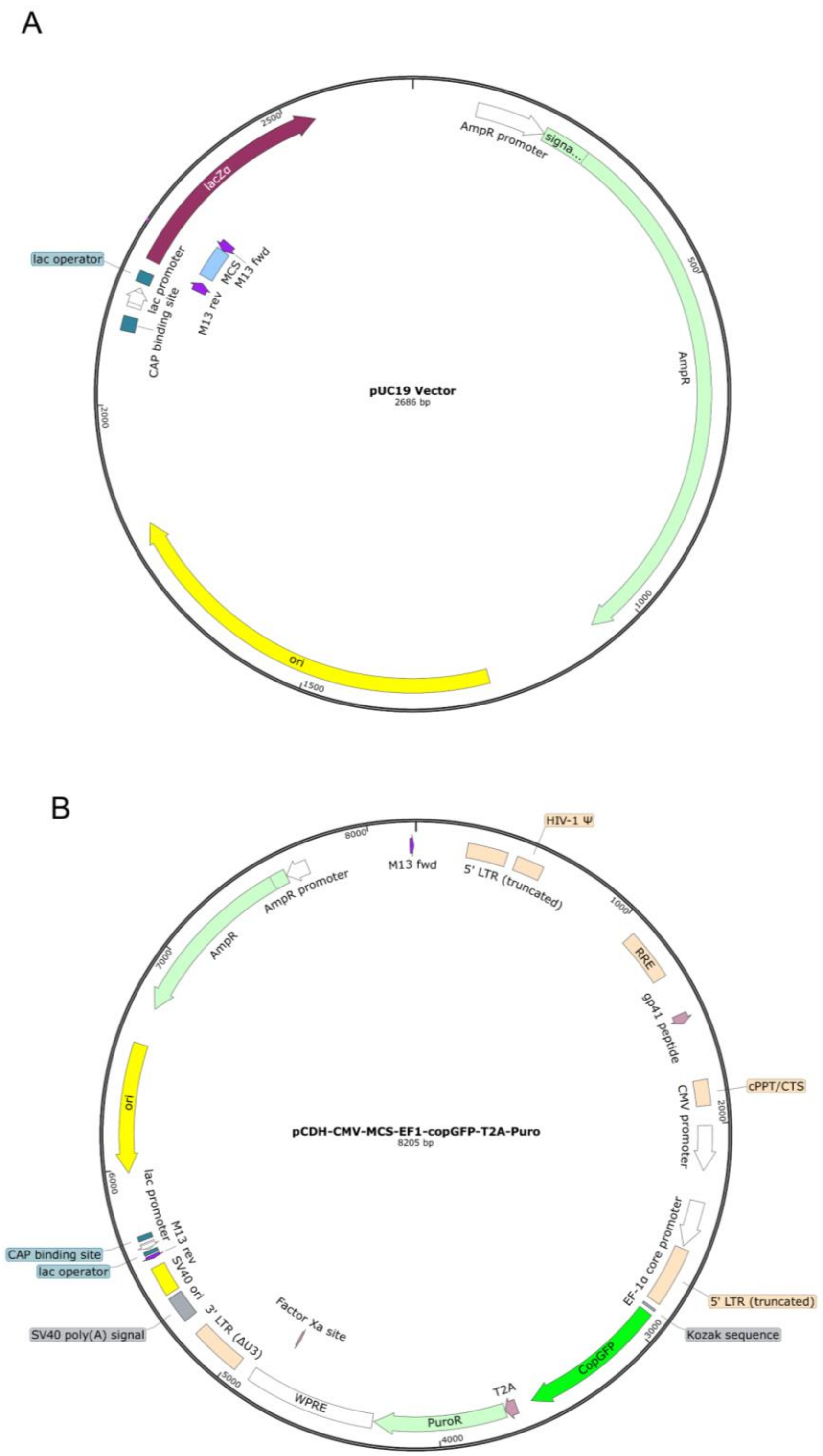

Fig. S1. Two wild-type plasmid DNAs employed in this study. (A) The pUC19 vector, (B) The pCDH vector with *GFP* gene.

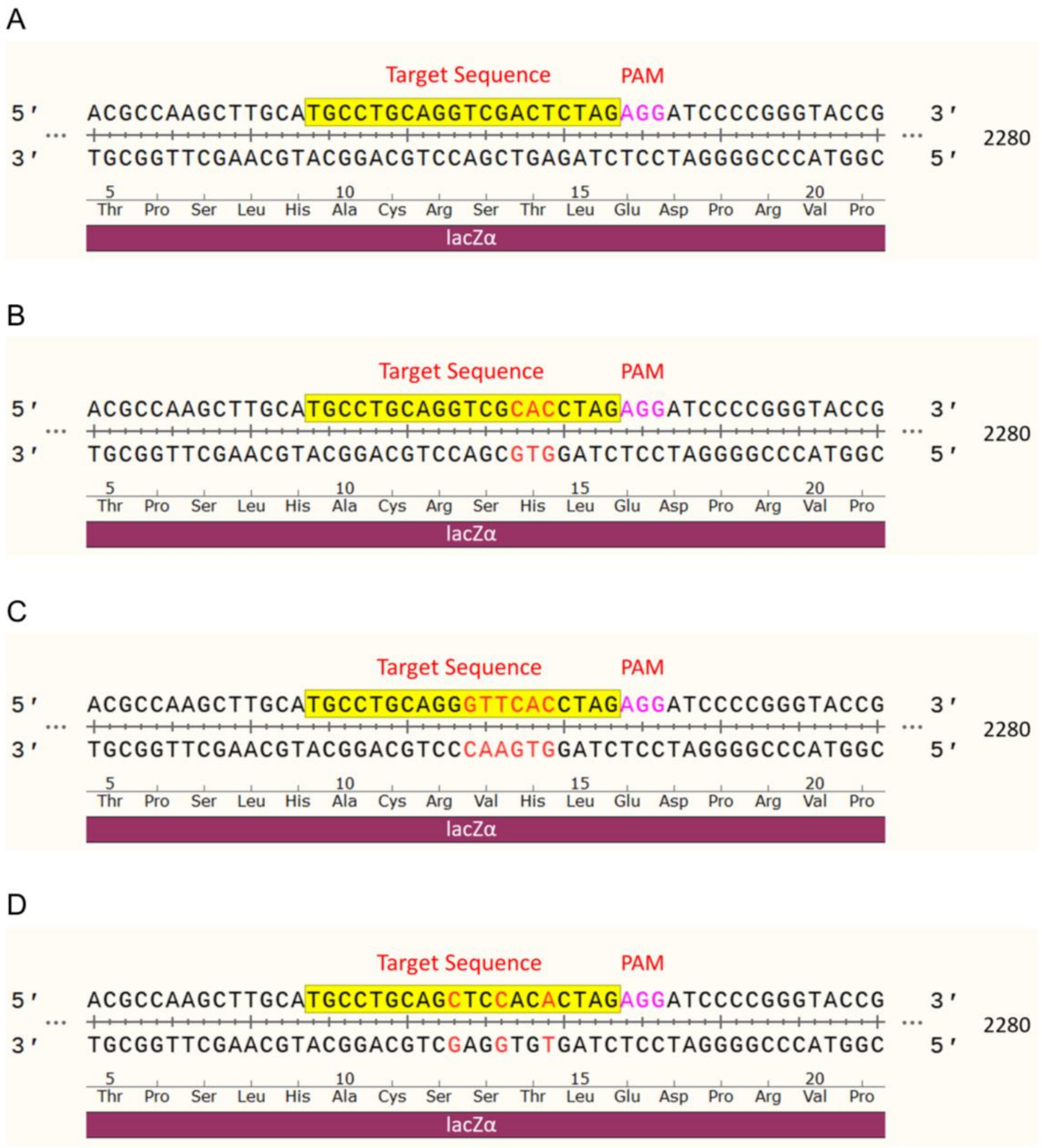

Fig. S2. Sequences of pUC19 plasmid mutagens in the *lacZα* gene region. (A) pUC19-Origin: the original sequence (gtcgact), (B) pUC19-Mut1: gtcgact→gtcgCAC, (C) pUC19-Mut2: gtcgact→gGTTCAC, (D) pUC19-Mut3: gtcgact→CtcCacA. The corresponding mutagenesis sites are marked in red, the protospacer adjacent motifs (PAM) are marked in pink, and the target sites are highlighted in yellow.

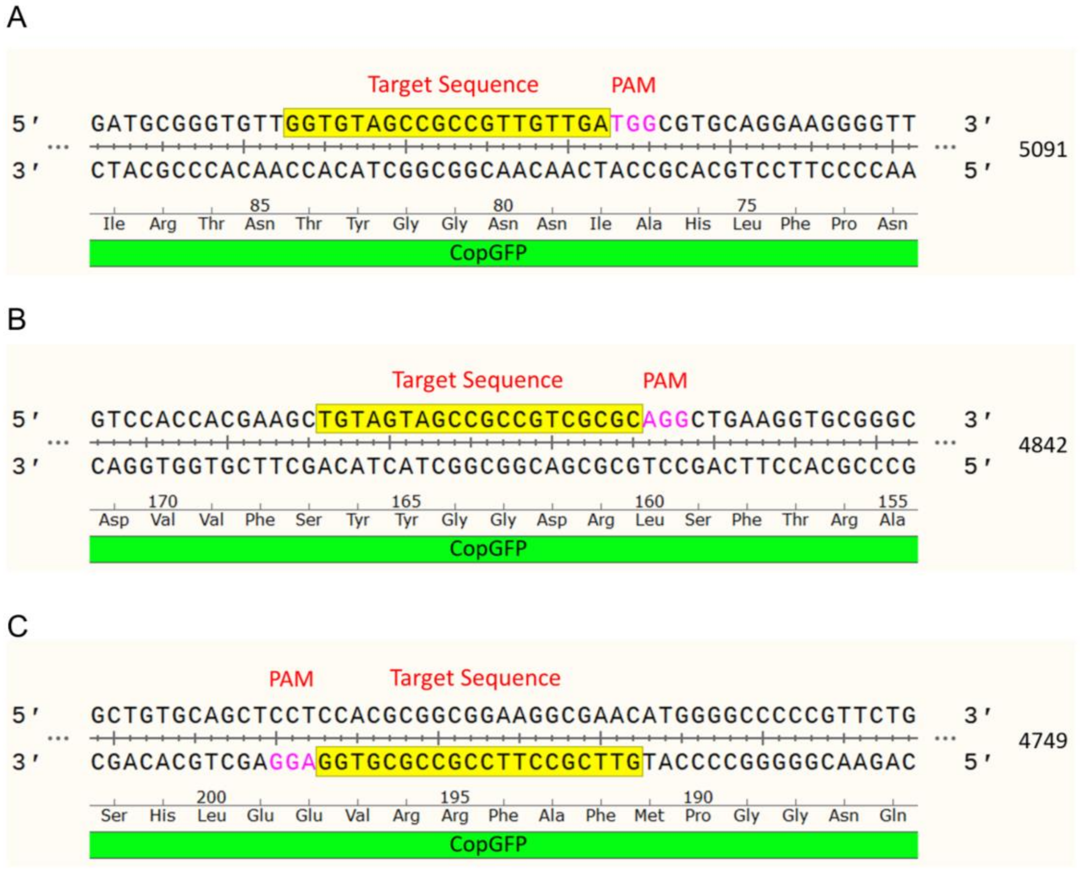

Fig. S3. Three target sequences of the pCDH vector in the *GFP* region. (A) pCDH-GFP-Pos1, (B) pCDH-GFP-Pos2, (C) pCDH-GFP-Pos3. The corresponding target sites are marked in red, the protospacer adjacent motifs (PAM) are marked in pink, and the target sites are highlighted in yellow.

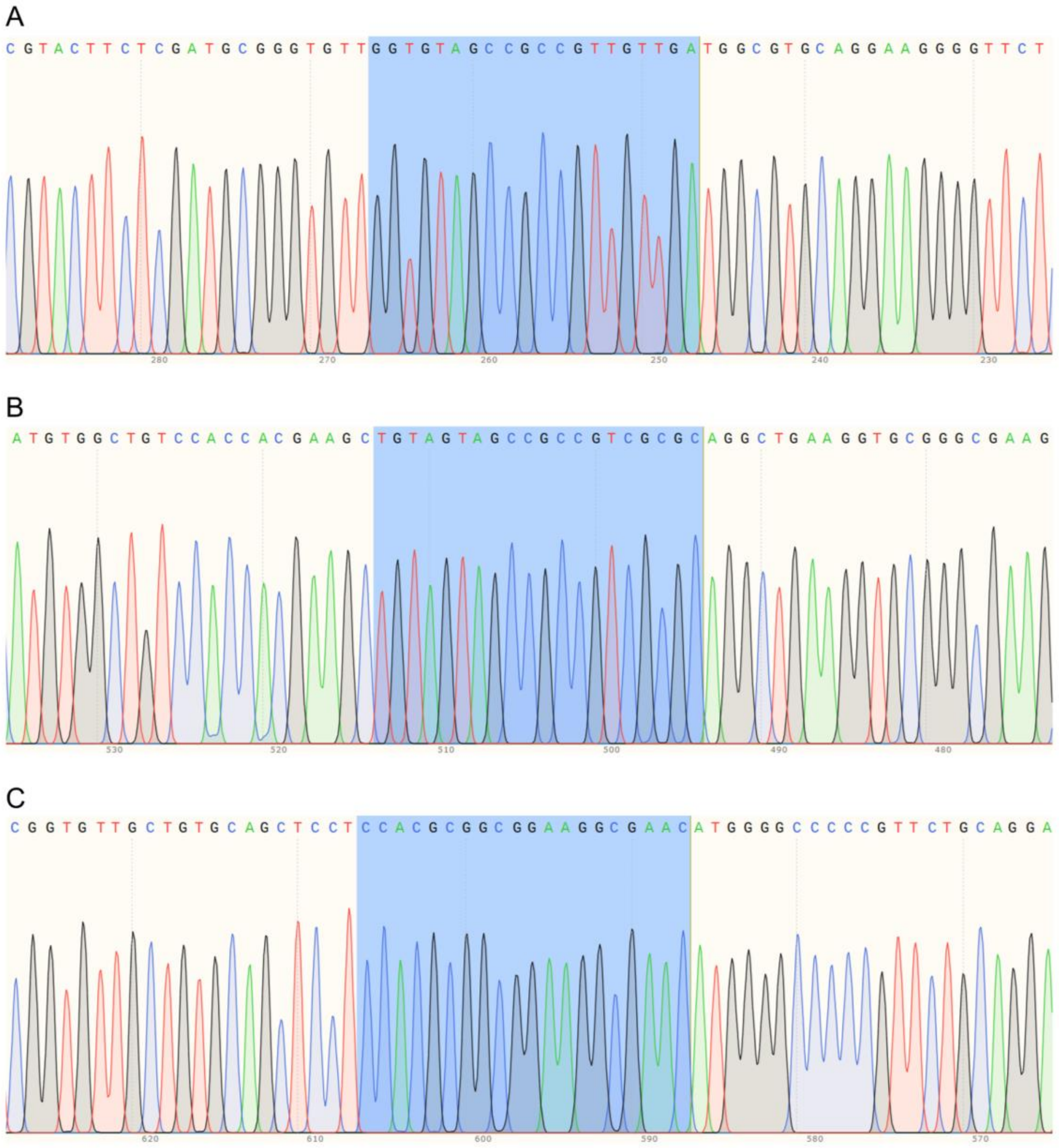

Fig. S4. Results of Sanger sequencing confirmed the three target sequences of the pCDH vector in the *GFP* region. (A) pCDH-GFP-Pos1, (B) pCDH-GFP-Pos2, (C) pCDH-GFP-Pos3

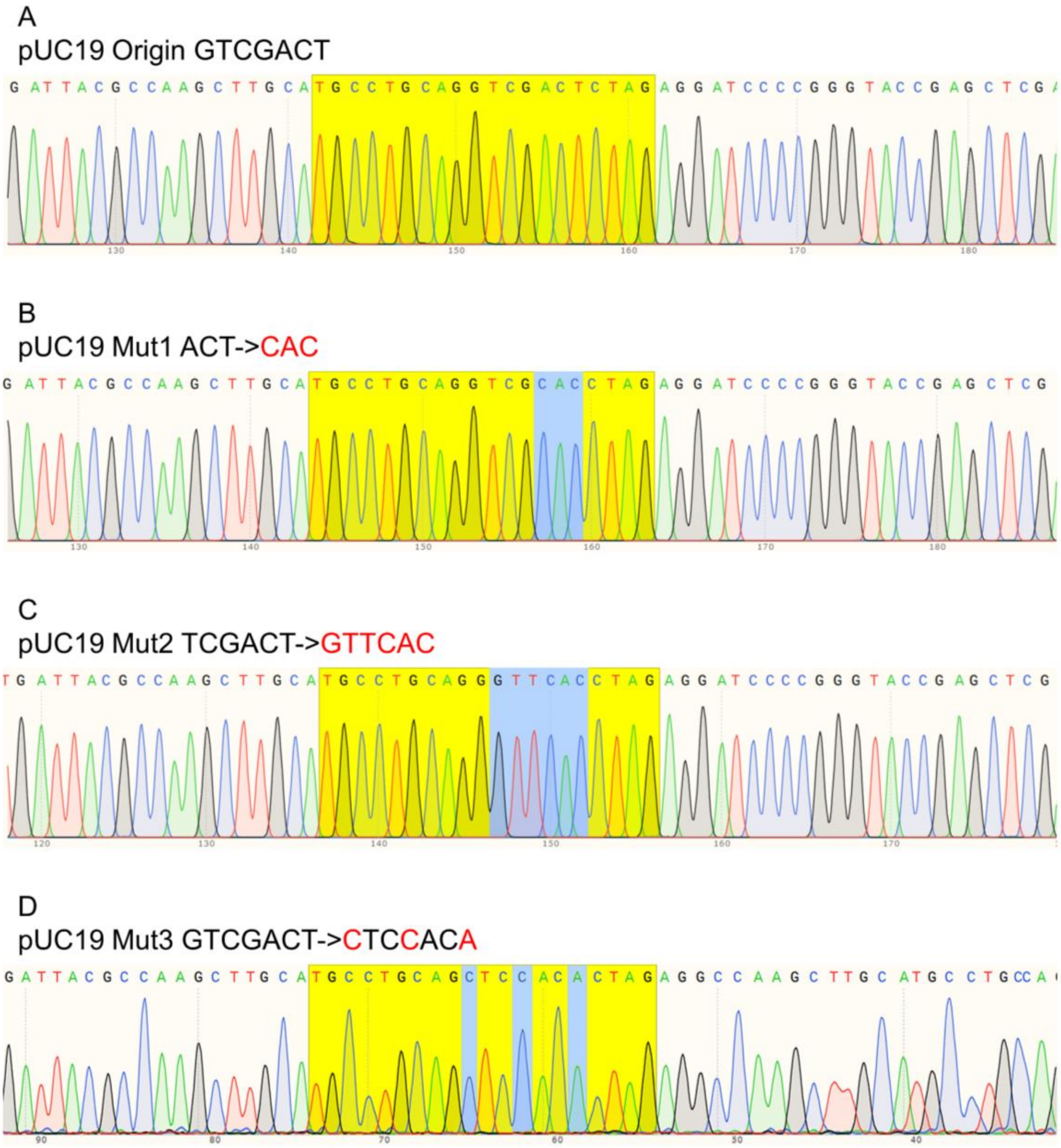

Fig. S5. Results of Sanger sequencing revealed that three recombinant gene sequences (Mut1, Mut2, and Mut3) in the pUC19 vector were constructed. (A) pUC19-Origin: the original sequence (gtcgact), (B) pUC19-Mut1: gtcgact→gtcgCAC, (C) pUC19-Mut2: gtcgact→gGTTCAC, (D) pUC19-Mut3: gtcgact→CtcCacA. The target sites are highlighted in yellow, and the corresponding mutagenesis sites are highlighted in blue.

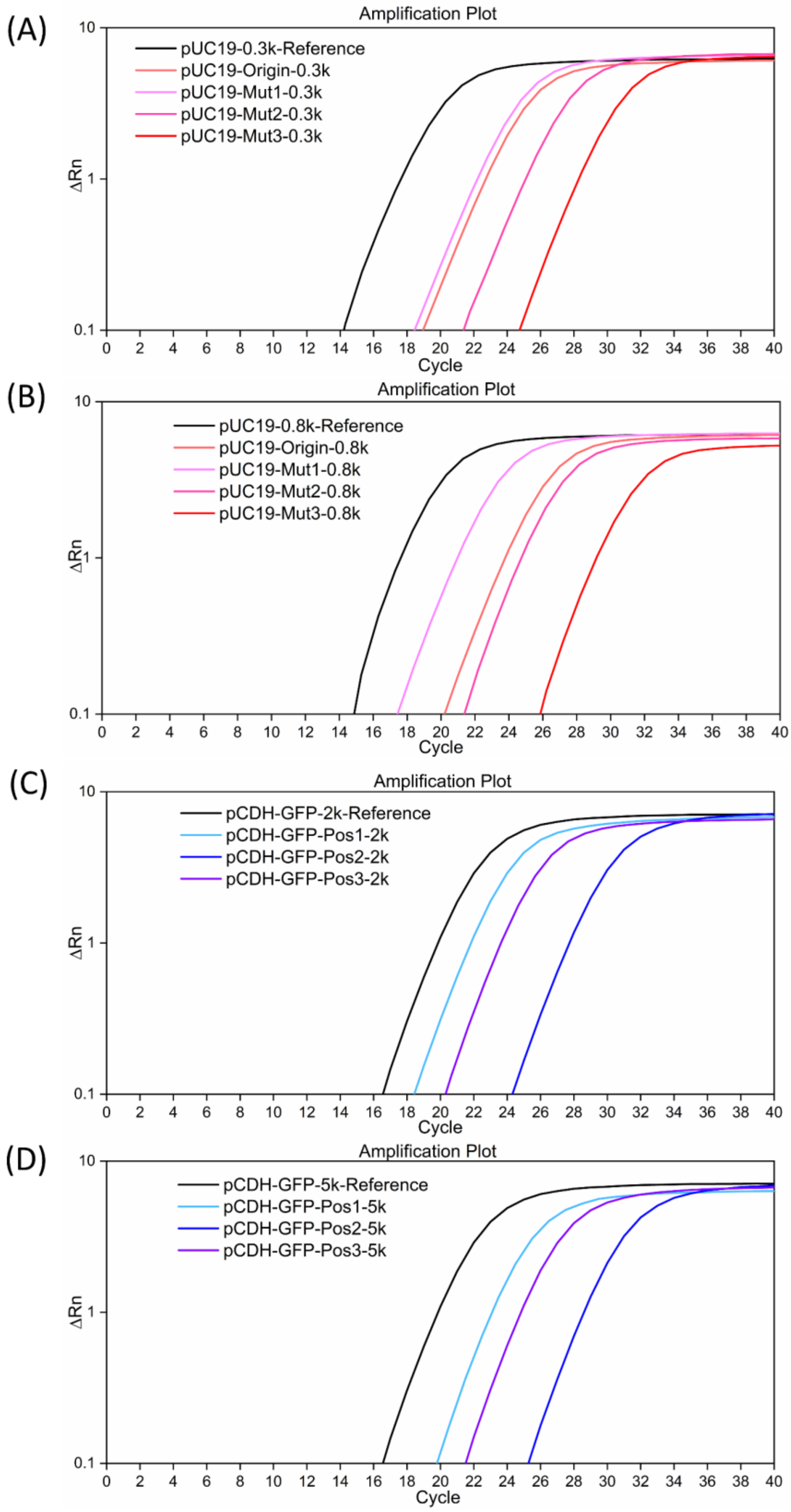

Fig. S6 qPCR analysis to determine the amount of target DNA samples captured by the dRNP-functionalized beads. (A) pUC19-0.3k, (B) pUC19-0.8k, (C) pCDH-2k, (D) pCDH-5k.

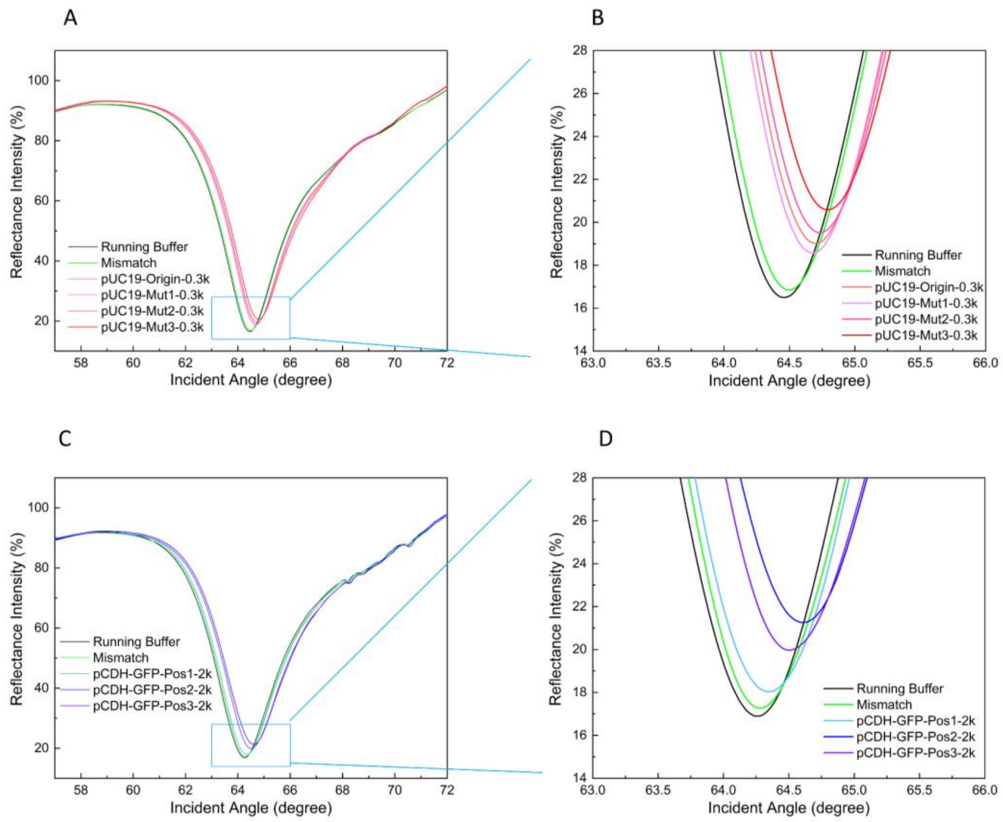

Fig. S7. SPR angular spectra of the dRNP-functionalized CRISPR-SPR-Chips detecting DNA fragments that contain different target sites. (A, B) Recombinant pUC19 amplicons of 0.3 kb detected by the corresponding dRNP-functionalized CRISPR-SPR-Chips (Origin, Mut1, Mut2, and Mut3). (C, D) The pCDH amplicons of 2 kb detected by the dRNP-functionalized CRISPR-SPR-Chips with different target sites (GFP-Pos1, GFP-Pos2, and GFP-Pos3).

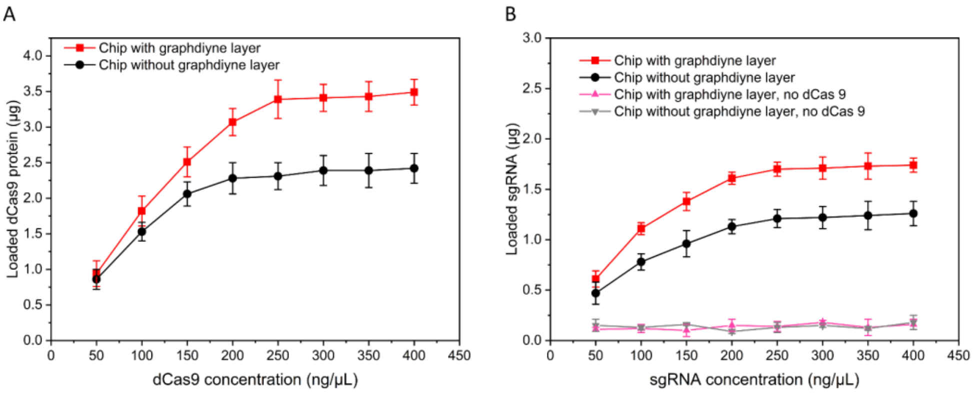

Fig. S8. Loading amount of dCas9 protein and sgRNA on the chips. After collecting all of the (A) protein or (B) sgRNA residue after incubation, the concentrations were measured to determine the amount of protein or sgRNA to be loaded on the chips. Calculated results of loading amounts of dCas9 protein and sgRNA on the Chips with or without a graphdiyne layer are present in these figures.

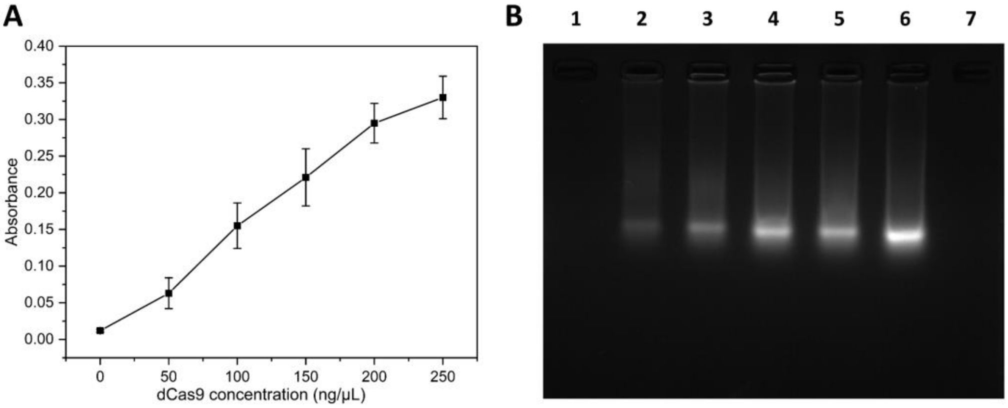

Fig. S9. Verification of the immobilization of dCas9 and sgRNA on the graphdiyne chips. (A) After immobilization using different concentrations of dCas9 protein solution on the graphdiyne chips, an immunological method was performed to verify the dCas9 on the chips (n = 3; error bars represent standard deviations). (B) After immobilization of dCas9 and sgRNA, the chips were further dealt with proteinase K to release the sgRNA combined with dCas9 and further identified using agarose gel electrophoresis. Lane 1: proteinase K solution but not incubated with the chips, no band appears on the gel; Lane 2-6: proteinase K solution incubated with chips previously immobilized with 50-250 μg/μL sgRNA, sgRNA was released and appeared on the gel; Lane 7: solution without proteinase K incubated with chip previously immobilized with 250 μg/μL sgRNA, no band appears on the gel. The data above illustrate both dCas9 and sgRNA were immobilized on the chips, and formed into the dCas9-sgRNA complex successfully.

Appendix C. Supplementary Tables

Target site	sgRNA sequence
pUC19-Origin	5'-GGGugccugcaggucgacucuagGUUUUAGAGCUAGAAAUAGCAAGUUAAAAUAAGGCUAGU-3'
pUC19-Mut1	5'-GGGugccugcaggucgCACcuagGUUUUAGAGCUAGAAAUAGCAAGUUAAAAUAAGGCUAGU-3'
pUC19-Mut2	5'-GGGugccugcaggGUUCACcuagGUUUUAGAGCUAGAAAUAGCAAGUUAAAAUAAGGCUAGU-3'
pUC19-Mut3	5'-GGGugccugcagCucCacAcuagGUUUUAGAGCUAGAAAUAGCAAGUUAAAAUAAGGCUAGU-3'

Table S1. The sgRNA sequences for the recombinant pUC19 amplicons. The corresponding mutagenesis sites are marked in red capital, and the target sites are highlighted in yellow.

Target site	sgRNA sequence
pCDH-GFP-Pos 1	5'-GGGGGUGUAGCCGCCGUUGUUGAGUUUUAGAGCUAGAAAUAGCAAGUUAAAAUAAGGCUAGU-3'
pCDH-GFP-Pos 2	5'-GGGUGUAGUAGCCGCCGUCGCGCGUUUUAGAGCUAGAAAUAGCAAGUUAAAAUAAGGCUAGU-3'
pCDH-GFP-Pos 3	5'-GGGGUUCGCCUUCCGCCGCGUGGGUUUUAGAGCUAGAAAUAGCAAGUUAAAAUAAGGCUAGU-3'

Table S2. The sgRNA sequences for the pCDH amplicons. The targe sites are highlighted in yellow, and -2k and -5k amplicons were use the identical primers for the same target sites.

Target site	sgRNA sequence
Exon7	5'-GGGUGCAUGUUCCAGUCGUUGUGGUUUUAGAGCUAGAAAUAGCAAGUUAAAAUAAGGCUAGU-3'
Exon47	5'-GGGUGCAUGUUCCAGUCGUUGUGGUUUUAGAGCUAGAAAUAGCAAGUUAAAAUAAGGCUAGU-3'

Table S3. The sgRNA sequences for the clinical DMD genomic samples. The targe sites of exon7 and exon47 are highlighted in yellow.

Mutagenesis site	Forward	Reverse
Mut1	5'-ccaagcttgcatgcctgcaggtcgCACctagaggatc-3'	5'-GTGcgacctgcaggcatgcaagcttggcgtaatcatg-3'
Mut2	5'-ccaagcttgcatgcctgcaggGTTCACctagaggatc-3'	5'-TCTGTGcctgcaggcatgcaagcttggcgtaatcatg-3'
Mut3	5'-ccaagcttgcatgcctgcagCtcCacActagaggatc-3'	5'-TgtGgaGctgcaggcatgcaagcttggcgtaatcatg-3'

Table S4. Primers for site-directed mutagenesis of pUC19, the mutagenesis sites are highlighted in red capital, and the PAM sequence is marked in orange.

Target sequence	Forward	Reverse
pUC19-0.3k	5'-CACAGATGCGTAAGGAGAA-3'	5'-CTACACCGAACTGAGATACC-3'
pUC19-0.8k	5'-GGCATCAGAGCAGATTGTA-3'	5'-GGAATTGTGAGCGGATAAC-3'

Table S5. Primers for PCR amplification of pUC19-0.3k and pUC19-0.8k. All the recombinant pUC19 vectors were used the identical primers for PCR amplification to obtain the amplicons with the same length.

Target sequence	Forward	Reverse
pUC19	5'-GTGTGGAATTGTGAGCGGATAA-3'	5'-GCAAGGCGATTAAGTTGGGTAA-3'

Table S6. Primers for qPCR analysis of all the recombinant pUC19 amplicons.

Target sequences	Forward	Reverse
pCDH-GFP-2k	5'-CCTGCTTGCTCAACTCTACG-3'	5'-GCTGCCTTGTAAGTCATTGGT-3'
pCDH-NoneGFP-2k	5'-GGTGCGAGAGCGTCAGTAT-3'	5'-GGAGCCAGTACACGACATCA-3'
pCDH-GFP-5k	5'-ATGCCGATTGGTGGAAGTAAG-3'	5'-GCTGCCTTGTAAGTCATTGGT-3'
pCDH-NoneGFP-5k	5'-CGCAGCAACAGATGGAAGG-3'	5'-CCGCTTAATACTGACGCTCTC-3'

Table S7. Primers for PCR amplification of pCDH-2k and pCDH-5k. Different amplification regions were selected to obtain the amplicons with and without the *GFP* gene.

Target sequence	Forward	Reverse
pCDH-GFP-Pos1	5'-GGCTACGGCTTCTACCACTT-3'	5'-TACTTCTCGATGCGGGTGTT-3'
pCDH-GFP-Pos2	5'-CAAGATCATCCGCAGCAACG-3'	5'-GTCCACCACGAAGCTGTAGTA-3'
pCDH-GFP-Pos3	5'-ACAGCTTCGTGGTGGACAG-3'	5'-TGAAGGCGTGCTGGTACTC-3'

Table S8. Primers for qPCR analysis of pCDH amplicons with different targe sites of the *GFP* gene.

Sample	ID	Description	Product	Source	Gender	Age at sample
H1	NA03349	Apparently healthy individual	Genomic DNA	LCL	Male	10
H2	NA03798	Apparently healthy individual	Genomic DNA	LCL	Male	10
H3	NA17508	Apparently healthy individual	Genomic DNA	LCL	Male	16
A1	NA05017	DMD exons 45-50 deletion	Genomic DNA	Fibroblast	Male	12
A2	NA05016	DMD exons 45-50 deletion	Genomic DNA	LCL	Male	12
A3	NA03929	DMD exons 46-50 deletion	Genomic DNA	LCL	Male	12
B1	NA05170	DMD exons 4-43 deletion	Genomic DNA	LCL	Male	9
B2	NA03780	DMD exons 3-17 deletion	Genomic DNA	LCL	Male	11
B3	NA03782	DMD exons 3-17 deletion	Genomic DNA	LCL	Male	11

Table S9. Information of clinical samples.

DNA analyte	k_a (10^5 $M^{-1}s^{-1}$)	k_d (10^{-3} s^{-1})	k_D (10^{-8} M)
pUC19-Ori-0.3k	0.077	9.8	127.27
pUC19-Mut1-0.3k	0.060	11.1	186.67
pUC19-Mut2-0.3k	0.083	9.2	110.84
pUC19-Mut3-0.3k	0.44	8.1	18.41
pUC19-Ori-0.8k	0.25	10.1	40.40
pUC19-Mut1-0.8k	0.15	11.4	75.86
pUC19-Mut2-0.8k	0.26	8.9	34.23
pUC19-Mut3-0.8k	1.05	8.0	7.61
pCDH-GFP-Pos1-2k	0.0082	8.3	1012.20
pCDH-GFP-Pos2-2k	1.19	4.2	3.53
pCDH-GFP-Pos3-2k	0.062	6.3	101.61
pCDH-GFP-Pos1-5k	0.019	8.2	431.57
pCDH-GFP-Pos2-5k	3.12	4.3	1.38
pCDH-GFP-Pos3-5k	0.11	6.5	59.09

Table S10. Values of kinetic constants determined for the interaction of CRISPR-SPR-Chip and DNA analyte.

Name	Limit of Detection	Time (min)	Pre-amplification	Detection System	Type of Analyte	Reference
SHERLOCK	2 aM	50	RPA	Cas13a and RNA reporter	RNA	[1] and [2]
HOLMES	1-10 aM	60	RPA	Cas12a and collateral ssDNA probe	ssDNA	[3]
HOLMESv2	10 aM	120	RT-LAMP	Cas12b and collateral ssDNA probe	RNA	[4]
CRISPR-powered COVID-19 Diagnosis	0.7 aM	50	RPA	Cas12a and collateral ssDNA probe	RNA (virus)	[5]
CRISPR-mediated DNA-FISH method	10 cfu/mL	30	No need	dCas9 and SYBR Green	DNA (bacteria)	[6]
Aptamer-assisted CRISPR-Cas12a	10 μM	25	No need	Aptamer, Cas12a and ssDNA reporter	ATP	[7]
CRISPR/Cas9 triggered ESDR	0.3 pM	85	No need	Cas9 and ESDR	ctDNA	[8]
CRISPR-chip	1.7 fM	15	No need	dCas9 and field-effect transistor	dsDNA (genomic)	[9]
Cas12a-CHA	1 fM	30	No need	Cas 12a and catalytic hairpin assembly	miRNA	[10]
CONAN	1 fM	30	No need	Cas 12a and scgRNA	dsDNA (genomic)	[11]
Ultra-localized Cas13a Assay	Single molecule	75	No need	Cas 13a and droplet microfluidic	RNAs	[12]
CRISPR-SERS	8.1 fM	70	No need	dCas9 and SERS	dsDNA (genomic)	[13]
CRISPR-SPR-Chip	1.3 fM	5	No need	dCas9 and SPR	dsDNA (genomic)	This work

Table S11. Comparison of nucleic acid detection techniques based on CRISPR system.

CRISPR: Clustered regularly interspaced palindromic repeats

RPA: Recombinase polymerase amplification

LAMP: Loop- mediated isothermal amplification

SPR: Surface plasmon resonance

ESDR: Entropy-driven strand displacement reaction

ATP: Adenosine 5'-triphosphate

CHA: Catalytic hairpin assembly

SERS: Surface-enhanced Raman scattering

References

[1] M. J. Kellner, J. G. Koob, J. S. Gootenberg, O. O. Abudayyeh, F. Zhang, *Nat. Protoc.* **2019**, *14*, 2986.

[2] J. S. Gootenberg, O. O. Abudayyeh, M. J. Kellner, J. Joung, J. J. Collins, F. Zhang, *Science (80-.).* **2018**, *360*, 439.

[3] S.-Y. Li, Q.-X. Cheng, J.-M. Wang, X.-Y. Li, Z.-L. Zhang, S. Gao, R.-B. Cao, G.-P. Zhao, J. Wang, *Cell Discov.* **2018**, *4*, 20.

[4] L. Li, S. Li, N. Wu, J. Wu, G. Wang, G. Zhao, J. Wang, *ACS Synth. Biol.* **2019**, *8*, 2228.

[5] Z. Huang, D. Tian, Y. Liu, Z. Lin, C. J. Lyon, W. Lai, D. Fusco, A. Drouin, X. Yin, T. Hu, B. Ning, *Biosens. Bioelectron.* **2020**, *164*, 112316.

[6] K. Guk, J. O. Keem, S. G. Hwang, H. Kim, T. Kang, E. K. Lim, J. Jung, *Biosens. Bioelectron.* **2017**, *95*, 67.

[7] C. Niu, C. Wang, F. Li, X. Zheng, X. Xing, C. Zhang, *Biosens. Bioelectron.* **2021**, *183*, 135907.

[8] M. Chen, D. Wu, S. Tu, C. Yang, D. J. Chen, Y. Xu, *Biosens. Bioelectron.* **2021**, *173*, 112821.

[9] R. Hajian, S. Balderston, T. Tran, T. DeBoer, J. Etienne, M. Sandhu, N. A. Wauford, J.-Y. Chung, J. Nokes, M. Athaiya, J. Paredes, R. Peytavi, B. Goldsmith, N. Murthy, I. M. Conboy, K. Aran, *Nat. Biomed. Eng.* **2019**, *3*, 427.

[10] S. Peng, Z. Tan, S. Chen, C. Lei, Z. Nie, *Chem. Sci.* **2020**, *11*, 7362.

[11] K. Shi, S. Xie, R. Tian, S. Wang, Q. Lu, D. Gao, C. Lei, H. Zhu, Z. Nie, *Sci. Adv.* **2021**, *7*, 1.

[12] T. Tian, B. Shu, Y. Jiang, M. Ye, L. Liu, Z. Guo, Z. Han, Z. Wang, X. Zhou, *ACS Nano* **2021**, *15*, 1167.

[13] H. Kim, S. Lee, H. W. Seo, B. Kang, J. Moon, K. G. Lee, D. Yong, H. Kang, J. Jung, E. K. Lim, J. Jeong, H. G. Park, C. M. Ryu, T. Kang, *ACS Nano* **2020**, *14*.
